# Spirit of the Foreign Periodicals, &c.

**Published:** 1838-01-01

**Authors:** 


### Spirit of the Foreign Periodicals, &c.

### Illustrations of Phrenology.

*Prefatory Remarks.* — In one of the recent numbers of this Journal, we gave an account of a discussion on phrenology, which took place a year or so ago at the Royal Academy of Medicine in Paris. That it attracted some notice from the friends of the science in this country, appears from the circumstance that it was transferred entire to the pages of one of our most respected cotemporaries.

Perhaps, however, the chief value of the report consisted less in the originality or perspicuity of the speeches which were delivered by the different members, than in the proof that was thus given of so many able men in the French metropolis having, of late years, assented to and embraced the fundamental positions of phrenology. The readers of the Lancet are well aware that M. Broussais has delivered an admirable course of lectures upon the subject, full of ability and enthusiasm, and on the whole exhibiting a pretty fair view of the present state of the science. He is one of the most active members of the Phrenological Society of Paris; and in addition to this distinguished teacher, we may mention the names of Andral, Bouillaud, Adelon, Ferrus, Amussat, and of several other distinguished physicians and surgeons in the French metropolis, as ranked among the friends and supporters of Dr. Gall's physiology of the brain.

Now, although we must confess that we are not inclined, on many occasions, to be very much biassed by the opinions and reasonings of our lively neighbours upon subjects which require much laborious and patient inquiry, we are nevertheless bound to admit that the authority of such men, as we have now mentioned, is more than sufficient to exact, if not assent to, at least an unprejudiced examination of, any doctrine which they view with favour. Independently of this consideration, the mere circumstance of Drs. Gall and Spurzheim's system having stood for so many years against the most vehement and determined, not to say the most scurrilous and unfair, attacks of wit, ingenuity, and malice, has been to our minds a sort of *a priori* argument, of no trifling weight, in favour of its probable truth.

It is due, however, to our readers that, as we now introduce the subject to their notice, we should confess that we are very far from being satisfied of the correctness of many of the positions which we find inculcated by almost all the professed advocates of phrenology up to the present day. There is an insufficiency of groundwork, a feeble and even flimsy mode of illustration, and withal so rapid and incautious a tone of decision upon not a few of the most uncertain details of the science, that we have been often utterly surprized at the unhesitating manner in which they have been announced and insisted upon. On this, as on many other subjects, the injudicious support of friends has been perhaps more injurious to the cause of truth than the open attacks of the enemy. To us it seems that the leading error of the phrenologists has been all along to assume as *proved* many details which, although they may be rendered probable by some observations, ought still to be considered rather as *queries* than *axioms.*

The only plan to arrive at any accuracy on these uncertain points is to examine the book of nature, by collecting exact casts of multitudes of characters, distinguished by strong and indisputable development of certain faculties. Until the science is studied in this manner only, and its disciples shew a more uniform spirit of caution, and more exact spirit of reasoning in all their inquiries, we much fear that they will long fail of making many converts among the students of philosophy. While, however, we think that there is much conjecture and mere fanciful speculation in many of the details of phrenology, we do not hesitate to assert that the fundamental doctrines of the system appear to us to be strictly in accordance with the truth.

If it be asked what we mean by the fundamental doctrines, we have no difficulty in replying thus; that the great divisions of the encephalon seem to exercise different functions in reference to the attributes of the mind;—that one part, the anterior, is intimately connected with the development and operation of the intellectual faculties; that the upper part is connected with the higher sentiments, or with such as are peculiar to man; and that the posterior and lateral portions are connected with the lower feelings, which are common to man and to animals.

We do not mean to say that we can point out the exact line of separation between each particular organ of these various powers or faculties of the mind. Much indeed has already been done by the labours of Drs. Gall and Spurzheim in this curious field of inquiry; and the very striking accuracy of many of their observations cannot, we think, be fairly gainsayed. For example, we deem it more than probable that the sexual propensities are associated somehow or other with the cerebellum; that the fierce, pugnacious, and revengeful feelings are connected with that portion of the cerebrum which is situated above and behind the ear; that self-esteem usually predominates in those persons in whom the crown of the head is lofty; that the higher principles of justice, benevolence, and of veneration, are generally more active and more powerful when the space between the forehead and the vertex is ample and large; that great breadth and expanse of the upper part of the forehead is almost always indicative of strong reasoning powers; and that the perceptive faculties of the mind are quick and energetic, when the lower part of the forehead, immediately above the eyebrows, is prominent and full.

The probability of some of these positions is very beautifully illustrated by many of the results of comparative anatomy. We know, for example, that there is a much greater proportional breadth and fulness of the cranium behind the ears in all carnivorous, than in any herbivorous animals. We have only to place the skull of a cat or dog beside that of a sheep and a hare to satisfy ourselves of the truth of this statement. We know of no exception to this law: it appears to be uniform, and therefore undeniable.

Again, if we compare the skull of a monkey with that of any other of the lower animals, are we not forced to acknowledge that the anterior part, the os frontis, is always much higher and more prominent in the former than in the latter? If this be the case, is it not reasonable to suppose that this configuration may very possibly be connected with the greater amount of intelligence, which all the ape tribe are endowed with?

Who is ignorant of the sagacity, intelligence, and attachment of the dog? and is not its anterior lobe always much fuller and larger than in any of the feline race, the characters of which shew how inferior they are in these attributes?

No candid person can deny the truth of these facts, however much they may feel inclined to question the accuracy of the conclusions, which we are inclined to draw from them. Many, we know, have been unwilling to yield their assent to any of the doctrines of phrenology, on the ground, as they allege, that they savour of materialism. But we have no intention to canvas this matter.* The truth of phrenology must be tried and tried only by a direct appeal to nature, or in other words by a cautious and accurate examination of the correspondence — if such actually exists—between certain physical developments and certain mental endowments. We have already alluded to the form of the cranium in certain tribes of the lower animals, as indicative of certain peculiarities of character and temper. The same, in our opinion, holds true in reference to our own race. That there is a natural and original difference

* There is a short article on this subject extracted from the French journal, La Phrenologie, in a subsequent part of this Periscope.

of disposition, feeling, and intellectual capacity in different children, before education can have at all swayed or biassed their characters, is, to us at least, as undeniable as that there is a diversity of outward feature and figure. Are not some hasty, irascible, and revengeful? are not others mild, inoffensive, and patient of injury? is not one selfish and self-confiding, and is not another full of affection, and all the kindlier feelings? Are not some quick to learn, apt to remark, and lively in all their little fancies and imaginings? and are not others of the same nursery slow, and dull, and thoughtful? Does not one child shew a marked taste or talent for music, another for drawing, a third for cleverness at handiwork, a fourth for mimicry, and so on?

If these instances will not satisfy the sceptic, we should then advise him to study his own character, and compare it with the characters of his friends and fellows. Does he not perchance feel, although he may be very unwilling to make the admission to another, that certain feelings, and propensities are by nature stronger and more impelling, while certain others are feeble and less active, in himself than in them? True; education, and self-control, and reflection may have taught him the necessity of encouraging the growth of one set, and of restraining the growth of another set of these powers. The very aim and object of every rational person is to do this. But still, in spite of all his efforts, has he not often occasion to regret the insufficiency of all his best endeavours either to stimulate the higher powers and sentiments of his nature, or to keep in check the lower? Does he not perhaps feel that, however much he may toil, and however ardently he may wish to equal some friend in the play of fancy, the eloquence of language, the perspicuity of thought, the beauty of benevolence, or the general evenness and equanimity of disposition, all his exertions are at best only partially successful? Yes; study and zeal will do a great deal: they may direct and strengthen, but they will never overpower or master Nature. Study will never make a mighty poet, or painter, or musician. Such men as Homer and Shakespeare, as Michael Angelo and Raphael, as Handel and Mozart, were *born*, not *made*, the true sons of genius.

It is quite unnecessary to say more on this subject—it does not, it seems to us, admit of dispute. Our opponents, however, may now ask, if we have any reason to suppose that the cerebral development of these men was more favourably organized than that of their fellow-mortals. We will not answer for more than one, as we do not remember having seen accurate busts of the others; but that one will suffice. It is that of our own immortal countryman. Who that has ever looked on that glorious expanse of brow in the head of Shakespeare, but has not felt that the portrait *must* be true of him, who stands so proudly eminent above all other minds? It is not possible to imagine this first and foremost intellect with a low, contracted, and shabby forehead: as well might you picture to yourself a Hercules with limbs of straw, or a Jove with a feather for a thunderbolt.

Equally true to this standard of form is the cerebral development of other great authors. View the head of Sir Walter Scott — what an astonishing, nay almost a disfiguring, altitude of the fore and middle parts. It almost seems as if the creative energy of his boundless fancy had been struggling for room, and the tenement had been too tiny for the inmate. Look too at the heads of Goethe, Cuvier, Byron, and Wordsworth—they are all prodigiously large and full over the anterior lobes of the brain; and why? because the intellects of these "giants of mind" are pre-eminently great and expanded.

But we have neither time nor scope to enlarge upon this subject. Some will perhaps be inclined to say that our remarks are vague and unsatisfactory; but by the candid mind they may, we think, be received with more favour, intended, as they are, merely as prefatory to the following illustrations, which we now proceed to submit to the reader's attention.

A work entitled "Caractères Phrenologiques," published by M. Baillière last year at Paris, was recently put into our hands. Although full rather of light gossip than of very instructive reading, we were tempted to make a few extracts from it, considering that they might be aptly enough introduced before the reports of some of the papers, which we had copied from the late numbers of "La Phrenologie," a new French journal, which is now incorporated in the Encyclographie des Sciences Medicales.

Our chief motive for introducing this journal to the notice of the English reader, on the present occasion, is rather to convey some idea of what is going on among our professional brethren on the Continent, than to express our approbation of its contents. We rather fear that they are not well calculated to reflect much credit on French phrenology, and its professors.

To return however to the "Caractères Phrenologiques" of M. Theodore Poupin, we may briefly state that he has attempted to illustrate the phrenological powers or faculties of the mind, by selecting an example of each from among the distinguished men of the present century, whose lives are on the whole well known, and which may therefore, he supposes, be fairly adduced in confirmation of his ideas.

He probably had in view the admirable, but unfinished, work of Dr. Spurzheim on "Phrenology in Connexion with Physiognomy." Would that M. Poupin's was worthy to be deemed a continuation of it!

But such as it is, we shall now give a brief analysis of its contents. The author commences with the lower or merely animal feelings.

*Amativeness.*—The reader is somewhat surprised to find that the example, which M. Poupin has selected of this propensity, is taken from Gall himself, "notre maitre."

We are not acquainted with the private life of the worthy Doctor; but M. Poupin assures us that he was a most devoted admirer of the "beau sexe." In describing however Gall's physiognomical and phrenological development, he does not say one word on the subject of his *amativité,* unless we are to interpret the expression, "on trouve autour de la bouche une expression indefinissable d'amour," to this effect!

*Philoprogenitiveness.*— The illustrations of this amiable feeling are preceded by some rather apt allusions to the more beautiful examples of paternal and filial affection, celebrated by the Greek and Roman poets.

M. Poupin thus regards the attachment of a child to its parent as springing from the same source with parental fondness; "for in general," says he, "that man is a good father, who has all his life been a virtuous son."

He then adduces those beautiful lines of Virgil, where Æneas addresses his aged father.

Ergo age, care pater, cervici imponere nostræ,

Ipse subibo humeris, nec me labor ipse gravabit;

Quo res cunque cadent, unum et commune periclum,

Una salus ambobus erit.

The example, which M. Poupin has selected of *philogeniture,* is that of M. Casimir Perier, the late prime minister of France. "What harmony, what unity, what justness of proportion in his portrait! We cannot say all that we think; but what energy in the expression of the nose alone!"

The character of M. Perier was certainly distinguished by great firmness and intrepidity of resolution; but these high attributes were associated, we are told, with the most devoted love of his children.

*Inhabitiveness.*—There are some men, says our author, who regard *habitivité* as a mere fanaticism; who will ridicule your attachment to the spot where you were born, to the play-ground where you romped, to the clock of your village church, to the stream that murmurs past it. But these must be cold and passionless beings, in whom self is all-predominant.

He then descants in exstatic language upon the love which all Frenchmen have for their native country. "When they shall cease to love France, there shall be no Frenchmen in France; there shall be no longer a France. La patrie! la patrie!—these shall be the last French words that we shall speak, when the world crumbles to pieces!!"

The extract, which is given from M. Saint-Pierre's affecting description of his reaching the shores of France after a long residence abroad, is very beautiful; but we cannot find space for it at present. It will remind the English reader of the charming introductory chapter of Washington Irving's Sketch Book.

Even the native of a large town retains a peculiar attachment to it, from its being the place of his birth and all his early recollections. Montaigne, speaking of Paris, says: "The more I have seen of other fine cities, the more does the beauty of this gain upon my affection; I love it tenderly, even to its very blemishes." All English readers must know that glorious description of Edinburgh in Marmion, so beautifully closed by these simple words,

"Mine own romantic town."

The feeling of attachment to the place of nativity is however always strongest in those parts of a country most remote from its great towns, and especially when the scenery of the country is bold, rugged and mountainous. Hence its force and power among the Bretons, Vendeans, the Swiss, the Irish and the Scotch.

M. Poupin gives a very affecting narrative of four Peruvians, who died at Paris three years ago, and in whom the recollection of their native mountains and forests seemed to have brought on a fatal melancholy. But the type "par excellence" of inhabitiveness is, in our author's opinion, Sir Walter Scott. He gives the reader a long extract from Washington Irving's recent work on Newstead Abbey and Abbotsford, in which the mighty minstrel is introduced as himself describing his love for the "grey bleak hills" of his native country.

M. Poupin seems to be well acquainted with English literature; for he goes on to speak of the characters in Lady Morgan's novels, O'Donnel, and Florence Macarthy.

The physiognomical expression of Sir W. Scott's face is quite indicative, we are told, of his character as an author of the most wonderful creative genius, and as a man of the purest and most benevolent affections.

*Attachment.*—The organ of this feeling, which is certainly much akin to the two last-mentioned, is situated rather higher up on the occiput, and somewhat more outwardly than inhabitiveness, and philoprogenitiveness. It is the fountain-spring of friendship, the prompting impulse of all the social affections. It has therefore a much wider range of action than either the mere attachment to place, or that to offspring.

M. Poupin seems to believe that "affectionivité" is very rare in the world. "When," says he, "it succeeds to love, it may be sincere, but its origin is not pure. Between women it is a phenomenon; between men it is not less rare. The great are too much occupied with their titles and their wealth to think much of friendship; the men of the world are too frivolous, and the men of wit are too vain to think about it." He then quotes the following lines from Fontaine:—

> "Chacun se dit ami, mais fou qui s'y repose,
> Rien n'est plus commun que le nom,
> Rien n'est plus rare que la chose."

He is willing however to admit that there are some, who are born for friendship.

Of all men in the present day, he thinks M. Lafitte most worthy of the distinction. His character is described as being full of all the kindly benevolent features of our nature, of strong attachments, of the noblest self-devotion, and of the most elevated sentiments.

*Combativeness* is the feeling which is next described, and which is illustrated in the character and by the phrenological development of the late General Lamarque.

Our author passes a very high eulogium on his memory. It closes in these remarkable words: "he died with the word 'Waterloo' engraved upon his heart. It was he who said, 'Let war come, and I shall measure myself with

Wellington! I have studied his tactics, I shall beat him!! Such was the fixed idea of this great General."

(Our readers will no doubt be pleased to learn what were the *intentions* of General Lamarque.)—*Rev.*

The remarks of M. Poupin on the organ of *destructiveness* are, it is to be hoped, not quite orthodox; as they bear rather hard upon us, knights of the lancet. For example, he adduces the late celebrated surgeon, Baron Dupuytren, as the illustrative example of this very unamiable feeling, and with great coolness observes that many medical men, like he, have selected their profession from the strong impulse of destructiveness! He then alludes to the horrid fondness, which some persons have to witness public executions; to the love of the English for cock and for prize-fights, of the Spaniards for their bull-fights, and of his own countrymen for the more ignoble, but not less disgraceful, combats of dogs and cattle, at the barriers, Villette and Chopinette, where not only the dregs of the lower orders, but sometimes also "d'elegantes et nobles dames" attend.

However humiliating therefore, says M. Poupin, the reflection may be, that there is in our nature an innate propensity to kill and destroy, the truth is forced upon us in spite of all our reluctance; and in no distinguished character can we find a more illustrative example of its existence—directed, we allow, to objects of usefulness and science—than in the late Baron Dupuytren, always excepting "le celebre docteur Roux!" In both, the organ of destructiveness is, we are told, very fully developed. If education and the higher feelings and faculties had not directed them to the cultivation of a pursuit like surgery, these two eminent men might have proved dangerous members of society!

[The reasoning of our author on the character of Dupuytren is very faulty. He, for one, may have had the organ of destructiveness large; but surely such an organization is not necessary to become an eminent surgeon.]

*Secretiveness.*—Old Tallyrand is selected as the representative of this very useful, but often very inconvenient (to others), feeling. It is the nursing mother of cunning, love of stratagem, hypocrisy, and lies, when uncontrolled and undirected by the higher sentiments and faculties of the mind. Under proper regulation, it is an admirable tactician; never disclosing our plans and wishes until the well-judged moment.

No one can go well through life, without the exercise of a certain degree of dissimulation. It is quite necessary to the *manege* of civilized life, to the courtesies and even to many of the suavities of modern society. Since the days of Machiavel every one has been accustomed to identify it with the very existence of those mighty gentlemen, the diplomatists. Hence it is we suppose, that Prince Tallyrand de Perigord, the high-priest of so many governments, is adduced as the representative of *secretivité.* The Italians have been long noted for the exercise of this quality. Whether however the phrenological organ is more prominent in them than in other nations, our author does not inform us. He does not even pretend to say that it is at all largely developed in the head of Tallyrand himself. He describes him, indeed, as a consummate master of political dexterity, and of all that train of courtier intrigue, which has formed too great a part of modern European diplomacy. Every one has heard of the statue-like imperturbability of his features. One of his countrymen has wittily remarked; "jamais un visage ne fut moins barometré."

*Acquisitiveness.*—This is a strong feeling in most men—the love of acquiring. It is the prompter to active exertion, in the merchant, the banker, the lawyer, and often too in the statesman. If inordinate and irregular, it encourages a spirit of avarice; if deficient, there is generally a prodigal and extravagant disposition. Paganini, the famous fiddler, is selected by M. Poupin as an example of the former vice; and as a type of a base *acquisité.* He abuses him as "le seul artiste avare de notre temps." From all that we have heard the Signor amply deserves this castigation. Our author tells us that he has so offended his countrymen by his abominable greediness, that he is for ever *chassé* from Paris.

We have nothing to say of the last of the propensities, *constructiveness*, and shall therefore pass on to the consideration of some of the higher feelings or sentiments.

It may be proper however to allude to the other two, more doubtful, propensities, *love of life*, and *alimentiveness*. In 1832 Dr. Spurzheim proposed to the Anthropological Society of Paris the recognition of a new phrenological organ, the seat of that instinctive attachment to life, which exists in all animals. This organ has been adopted by Professor Dumoutier. It is supposed to be situated at the base of the brain immediately above the cerebellum, and corresponds externally with the mastoid process of the temporal bone. Our author has very strangely selected as the "tableau vivant" of this propensity, the late Leopold Robert, a young Parisian painter of great promise, who put an end to his existence from mere ennui!!

The other propensity, *alimentiveness*, the organ of which is said to be situated immediately in front of the ear, is exemplified by the portrait of a venerable most judge-like person, M. Henrion de Pensey, who, we are told, is justly celebrated in the records of gastronomy, and who told Laplace one day that he regarded the invention of a new dish as more interesting than the discovery of a new star!

We should suppose that M. Poupin himself is quite a connoisseur. "Gourmandise," says he, "paid the enormous burden imposed by the enemy in France in 1815. Barbarous as they were, they could not withstand the attractions of our restaurans. Paris became one vast *salon à diner*, where these bearded savages gorged themselves upon the fine meats, fish, pastry, and wines of our metropolis. The money thus spent was enormous. These were the days when Very, Achard, Beauvilliers, Sullot, &c. made immense fortunes."

*Self-Esteem.*—Our author descants eloquently upon the excellence of this feeling, when under proper control. Broussais, "le plus grand novateur et le premier medecin de notre siecle," is a pattern of it to perfection. His portrait therefore is selected to adorn the commencement of the chapter. He is said to be very like Gall; and M. Poupin describes in glowing terms his many excellencies. Not one word, however, is said either of his cerebral development, or whether the organ of self-esteem is large on his cranium, or not. It appears to be taken for granted.

*Love of Approbation* is large, we are told, in the character of M. Scribe, the prolific author of vaudevilles, and light dramatic sketches; and *Caution* in that of M. Dupin, the able advocate, and the accomplished statesman. Some of the remarks of M. Poupin on the working of this feeling are apt and good. "When it is inordinately developed, it engenders irresolution, melancholy, and hypochondriasis; if combativeness be at the same time small, the character is pusilanimous and even cowardly. The irresolute man—he without character, without energy, without a will—goes on usually from one fault to another, from regret to regret, a victim of complaisance, the dupe of good-heartedness, the sport of his associates, who does not get much credit for his virtuous actions, and to whom all the evil, that he can do, is charitably imputed." His description of the chaiacter of M. Dupin, the elder, is in the following words:—"Look at this forehead, full of grand and solid ideas, the eye which pierces the surface of objects, the expression of taste and elegance which reigns around the mouth, and especially the *ensemble* of that face where Nature has written, in physiognomical characters, *Caution* and *Dexterity*. Observe at the same time the horizontal position of the eyes, indicative of calm confidence, and it is impossible not to recognise the features of M. Dupin."

[This is all very flimsy and moreover far from being applicable to the portrait in question. There is a sad incongruity between the eloge of the author and the drawing of the artist.]

*Benevolence* is exemplified by the head of M. Beranger, so much admired for his beautiful odes and chansons, and whom our author panegyrises as one of the most noble, and the most pure creatures which has ever existed. After passing a high encomium on him as a man, and as a writer, he rather prettily adds, "Beranger est le type de la vraie sagesse. Chez lui c'est bien plus le cœur qui pense que la tete." No one, who is in the habit of attending in the most superficial manner to character, can for a moment hesitate to admit that there is a striking difference as to the benevolent attribute of different persons. M. Poupin has repeated the oft-told anecdote of the famous Turenne. The great general was leaning over the balcony of his window, dressed in a loose vest, and with a plain cotton cap on his head. His valet, mistaking him for one of his fellow servants, gave him a hearty slap on the back. Turenne turned round, rather surprised at this familiarity: the valet, confounded, fell at his feet and beseeched forgiveness for his error: "And if it had been Jacques the cook," said his master coolly, "there was surely no occasion to have struck him so hard."

A more exalted instance of benevolent feeling is that told of the virtuous Fenelon, who inculcated the doctrine of toleration on his pupil the Duke of Burgundy, in these memorable words, "Suffer all forms of religion, since God himself suffers them."

The next phrenological sentiment to be noticed is *Veneration*. M. Lamartine, the distinguished traveller, is adduced as an instance of the large development of this organ. It is very full also in the heads of M. M. Chateaubriand and Beranger. This is the feeling that leads the mind to adore and venerate not only the invisible Supreme Being, but all who are exalted above us either by station and talents, or by the claims of affection and love.

M. Poupin quotes the following passage from M. Lamartine's Voyage en Orient, as beautifully illustrative of his character.

"To explain to myself how, at the close of my youth, at that epoch of life when man withdraws himself from the ideal world to enter upon the world of material interests, I have left my lovely and peaceful life at Saint-Point, and all the innocent delights of a domestic fireside which is blessed by a wife, embellished by an only child, I say to myself —*this pilgrimage*, if not of a Christian, at least of a man and of a poet, *would have so pleased my mother*. This voyage of a son, whom she so tenderly loved, must please her even in the celestial abodes where I now see her; she will watch over us, she will place herself, like a second Providence, between us and all the storms of life, between me and the simoon, between me and the wild Arabs of the Desert! She will protect from every danger a son, an adopted daughter, and a little grandchild, the visible angel of our destiny; and, if there be imprudence in this my enterprize, she will obtain pardon for me above, in virtue of the motives which urge me—love, poesy, and religion."

The sentiments of *firmness, conscientiousness*, and *hope*, are illustrated by the portraits of, and by remarks on, the characters of Boissy d'Anglas, Chateaubriand, and Silvio Pellico.

*Marvellousness* is exemplified in the German poet, Hoffmann—a writer of great genius, but one, whose love of the supernatural was apt to lead him into extravagant excess. He was constantly haunted with the dread of some undefined danger, and his nights were continually disturbed by spectres and horrible phantoms, the creations of his own wild fancy.

He was small of stature, of a highly nervous temperament; his look was wild and almost savage; his hair was black. His cat Murr, of diabolic memory, was always at his side, and he was almost continually smoking. All these minutiæ are admirably represented in his portrait, which seems to have been hit off at a moment when he was writing these words—"Why do my thoughts, whether I am awake or asleep, always tend, in spite of all my efforts, to the gloomy subject of insanity? It seems to me as if I felt my disordered ideas escaping from my mind like hot blood from a wounded vein."

Hoffmann was an example of marvellousness abnormally developed, and morbidly excited at the same time.

*Ideality* is portrayed in the head of Victor Hugo, one of the leading novelists of the modern French school. Some of the remarks of our author on the attributes of this sentiment are very good. For example: "Ideality is the noble spring of all genuine enthusiasm and of all exalted feelings; it is, so to speak, genius materialised; it enlarges and dignifies the soul, and widens the boundaries of the *possible*. It is this secret, profound, and irresistible impulse that spurs on the soul to all its loftiest and purest efforts; that fires the poet and the patriot, that exalts the conceptions of the painter and sculptor, that breathes the 'witchery of sweet sound' over the soul of the musician, and that even animates and sustains the virtuous mind to many of its best deeds, and many of its holiest purposes."

*Gaiety* or *mirthfulness* is strongly developed in the head of that brilliant wit of Paris, M. Jules Janin. He therefore is adduced as the type of this faculty. His character is summed up in these words: "il est jovial, vif, animé, bon garçon, abandonné, sans façon, gai, joyeux, heureux." He is happy at nothing, but often as unhappy, he knows not why; passionate and enthusiastic about trifles, cold and disdainful in the midst of most important events; surrounded with enemies and rivals, yet quarrelling with or disliking none; apparently quite unconcerned about the annoyance or mischief he may cause to others, and as indifferent to what they may do to him; self-indulgent, *facile à vivre*, and often having nothing which he can quite call his own; a man of luxury, and who is yet satisfied with the most humble conveniences; in short an excellent good person at heart—such is M. Janin.* He has been a most prolific writer for the daily journals, and his pen has been engaged in most of the literary enterprises of the age. He is the author of several novels and tales. In fine, the character of this strange creature is an "improvisation every hour; it is the journal of each day. He represents journalism as M. Lamertine represents the Ode, and Victor Hugo does the drama of the present day."

We come now to allude briefly to the Intellectual faculties, and of these, the Perceptive are very properly treated of first.

*Individuality.*—This organ is largely developed in the head of M. Orfila, whose portrait therefore heads the chapter. This distinguished chemist and physician is well known to our profession, as he that has contributed most to the advancement of medical jurisprudence. His unwearied researches on this interesting department of science prove, most indisputably, how acute and vigorous his mind is in the observation of phenomena; and this character is strikingly in accordance with his phrenological organization.

*Form*, is exemplified in the portrait of Baron Gros, the celebrated painter of the battles of Arcola, Jaffa, Eylau, &c. and of the dome of the church of St. Genevieve; *size*, in that of M. Arago; *weight* or *resistance* in that of Baron Dupin; *colour* in that of Delaroche, the painter; *locality* in that of Jacquemont, the author of Travels in the East; *number* in that of M. Ampere; *order* in that of the immortal Cuvier; *eventuality* in that of Andrieux, a celebrated professor of French literature; *time* in that of Lablache, the Coryphæus of singers, *il premier cantatore du monde, la heros du chant; melody* in that of Rossini; and *language* in that of the famous oriental scholar Baron de Lacy.

Our limits prevent us from entering into more minute details of the different faculties now enumerated, or of the characters which are adduced to illustrate them.

The only remaining faculties, which remain to be mentioned, are those which have been denominated the reflective; viz. *comparison*, and *causality*. The former is said to be very largely developed in the head of M. de la Mennais, author of the Essay on Indifference in Matters of Religion; and the latter, in M. Geoffroy Saint Hilaire, the celebrated anatomist and philosopher.

We must now take leave of M. Theodore Poupin and his "Caractères Phrenologiques." He has afforded us some amusement, if not a great deal of instruction. There is a light-hearted vivacity about his work, and withal a tone of gentlemanly feeling and literary taste which may go far to recommend it to his countrymen, and encourage them, for the mere nonce, to attend to the curious subject of which it treats. Even the English phrenological reader may be pleased with its perusal—there are passages here and there which will well repay the trouble. Before, however, quitting the subject of physiognomical phrenology, we cannot deny ourselves the pleasure of alluding to a masterly work on this curious enquiry by the late Dr. Spurzheim himself, and which, we are surprised to find, is not much known in this country—at least far less known than it deserves to be. It is certainly the most attractive and popular of all his writings, and withal as thoroughly imbued with his philosophic cast of thought as the most profound of them—we allude to his "Phrenology in connexion with the Study of Physiognomy," illustrated with admirable drawings of thirty-four portraits of distinguished individuals.

After some prefatory remarks, very sketchy and full of apposite quotations, on the interest which all people must feel in studying physiognomy, and on the proper method to be followed in doing this, he gives a most scientific description of the physiognomical signs, first of the body, then of the face or features, and lastly of the entire head.

In the first chapter, on those of the body, there are some excellent remarks on the difference of temperament in different individuals, as influencing their physical as well as their mental attributes.

He adheres to the old quaternary division into the phlegmatic, the sanguine, the bilious, and the nervous temperaments, each being illustrated by a very graphic delineation—but very justly remarks that it is rare to find any one of them pure and unmixed. "They are mostly found conjoined," says our author, "and occur as lymphatic-sanguine, lymphatic-bilious, sanguine-lymphatic, sanguine-bilious, sanguine-nervous, and so forth. The individual temperaments, which predominate, may be determined, but it is difficult to point out every modification."

The influence, which the temperament exercises on the character of the individual, is thus very faithfully pointed out: "It is important, in a physiological point of view, to take into account the peculiar constitution or temperament of individuals, not as the cause of determinate faculties, but as influencing the energy with which the special functions of the several organs are manifested.

Their activity, generally, is diminished by disorder in the functions of vegetative life, and it is favoured by the sanguine, and still more by the nervous temperament."

In the chapter on physiognomical signs of the face, Dr. Spurzheim, while he shews the fallacy of trusting alone to these in the detection of the character, (as Lavater and his disciples have done,) shews with great truth that "certain forms of face, and certain expressions of features agree better than others with certain characters." "Nevertheless," adds he, "it remains certain that the same character is to be observed in conjunction with very dissimilar faces, and that the character by no means depends on the configuration of the face, although the face and character harmonize, just as do all the parts of a good picture."

On the national peculiarities of face, the following memoranda will be read with interest.

"The Jews, though they have been dispersed over all the countries, and have lived in all the climates of the globe for many centuries, still preserve a particular and distinguishing physiognomy. Peculiarities even mark the two tribes of Judah and Benjamin; in that of Judah, for example, the face is round and the cheeks are prominent; while in the tribe of Benjamin the face is lengthened, the cheeks are but slightly prominent, the nose is acquiline, and the eyes are lively."

Treating of our own country-people, Dr. Spurzheim says:

"England and Scotland having been occupied by various tribes, particular districts of each have a population originally different. In the County of Norfolk the same round and well-fed figures are seen, which Rubens has transferred to his canvas from the natives of Holland. On the borders between Scotland and England, the Roman form of face is still found. In the South again, the Saxon face is most common."

It is however chiefly in the third chapter, that which illustrates the physiognomical signs of the entire head, that the reader will be most pleased with the remarks of our author—they are so apt, so original, and ingenious, and *apparently* so very just and confirmatory of the general principles of phrenological science. We have no intention to enter upon particulars at present; neither our limits nor our time will permit this. But we may briefly observe that the precepts, which Dr. S. has so perspicuously laid down and illustrated in this section of his work, will deserve the careful attention not only of the enlightened traveller and of him that is curious in matters of natural history, as well as the painter and sculptor, but also of the philosophic physician and of the man of general science, who feels a pleasure in studying Nature's operations, in the noblest department of her works, the development and exercise of the human mind.

The mutual influences of the propensities or animal feelings, the sentiments or more exalted feelings, and the intellectual faculties,—these sometimes antagonizing, at other times beautifully according with, each other—are illustrated with great perspicuity and force.

This is the strictly metaphysical part of the disquisition, and it is altogether excellent. Whether our author is equally successful in shewing that the strength of these various powers of the mind is, in all cases or even generally, proportionate to the size and development of certain parts of the cerebral mass, and whether they may be measured by an inspection of the cranium outwardly, we are not prepared to say. This, in short, is the sum and substance of phrenology; and we have already said that we have not attended to the subject sufficiently to warrant us in giving an opinion of our own.

This only we shall say, that if any one wishes to acquire a fair and luminous view of the general principles of phrenology, he will do well to peruse this work of Dr. Spurzheim, before entangling himself in the more minute and elaborate details of any regular treatise. It is certainly the book which the true friend of the science will put first into the hands of a disciple.

The historical sketches of the different characters—including Caracalla and Nero, Seneca and Zeno, Cicero and the Roman Gladiator, Popes Gregory VII., Martin V. and Pius VII., Richelieu and Walsingham, Henry IV. of France and Sully, Charles XII., and Catharine II. of Russia, Danton and Malasherbes, Drs. Priestley and Price, &c.—are, on the whole, executed with perfect impartiality and truth. To each memoir is prefixed a short statement of the phrenological development of the different characters, such as is observed in their respective portraits.

As a specimen of the general style of this part of the work, we select the description of the heads of Nero, and of Cardinal Richelieu.

"This figure (of Nero) is after an antique bust in the Royal Museum at Paris. The forehead is low, and the whole sincipital region small; the organs of veneration and benevolence are particularly defective, while those of firmness, self-esteem, and of all the animal propensities are very large. The basilar and occipital regions are greatly superior in size to the upper and fore parts of the head. In whatever situation such a cerebral organization is placed, the animal nature will be apt to overpower the peculiarly human sentiments." It is unnecessary to allude to the actual life of this monster of iniquity.

And now with respect to Richelieu, the all-powerful minister of the French Court, Dr. Spurzheim remarks:

"The forehead of this portrait, particularly in the region of the perceptive faculties, is large, and the width of the head generally is greater than its elevation. The organs of acquisitiveness, secretiveness, destructiveness, firmness, self-esteem and love of approbation are strongly marked; while those of benevolence, veneration, and consciencious-ness are small. Such a man will be talented, but artful; he will be guided by selfish motives rather than by love of truth; religion in his hands will be but a means of gaining his immediate ends, and by gratifying his worldly intentions. He will sacrifice his enemies without pity or remorse; and in every situation, as father or as husband, as head of the church or of the civil government, he will insist upon being obeyed."

As a *set-off* to these two unamiable portraits, let us hear Dr. Spurzheim's delineation of an elevated character.

There is none better suited for our purpose than that of Melancthon, who is universally represented, by Catholics as well as by Protestants, as one of the highest ornaments of the Christian Church.

"The organization of this head differs widely from that of Luther. It is very narrow above and behind the ears, and the whole basilar region is very small; almost the whole of the brain indeed lies in the frontal and sincipital regions, both of which are exceedingly large. It is the brain of an extraordinary man. The organs of the moral and religious feelings predominate greatly, and will disapprove of all violence, irreverence, and injustice. The forehead betokens a vast and comprehensive understanding: the *ensemble* a mind the noblest, the most amiable, and the most intellectual that can be conceived. If there be any thing to regret, it is that the organs of the animal powers should have been so small in comparison with those proper to man."

In closing our notice of Dr. Spurzheim's work on "Phrenology in connexion with Physiognomy," we cannot refrain from once more very earnestly recommending this most ingenious and instructive work to the notice of our readers.

---

### CHARACTER OF CUVIER.

"In almost all the portraits of Cuvier, large or small, that forehead, which has given birth to one of the sublimest theories in science since the days of Newton, is observed to be not so much developed as might have been expected; and yet, notwithstanding this physiognomical defect, how wonderfully expressive it appears!

From the summit of the head to the neck, from the arch of the forehead to the chin—setting aside every other mark—what may we not anticipate from such an organization? Yes; it is thus that the Almighty wills to form the mighty sons of genius*—of such a genius as that which, by the sole aid of

---

* The following extract from the amusing work Peter's Letters to his Kinsfolk, although not strictly applicable, will be read with interest.

"In the general form of Sir Walter Scott's head, so very high and conical, and, above all, in the manner in which the forehead goes into the top of the head, there is something which at once tells you that there is lofty enthusiasm and passionate veneration for greatness, which must enter into the composition of every illustrious poet. In these respects Scott bears some resemblance to the busts of Shakespeare, but a much closer resemblance to those of Corneille; and surely Corneille was one of the most favoured of all poets, in regard to all that constitutes true poetic soarings of conception.

No minor poet ever approaches to this conformation; it is reserved for 'earth's giant sons' alone. * * * I do not think that the head is so long from stem to stern as Lord Byron's, some fragments dug out of the bowels of the earth, recomposed, as it were, creatures which had fallen into nothingness, and which constructed anew from a few broken pieces an enormous edifice, to shew to us poor beings what the earth once was, what it is now, and what it probably will become at a future period.

We say nothing of the eyebrow, of the expression and form of the eyeball, and of the contour of the nose; although these signs, according to Lavater, are indications of a profound and exquisite judgment, of an invincible industry, of a singular aptitude for inquiry and observation, a most scrupulous regard for order and arrangement, and, in fine, an immoveable mind, gifted rather with solidity of thought than brilliancy of fancy, rather with depth and firmness than a delicate sensibility of feeling.

The numerous works of the great naturalist bear witness to the mighty powers of his mind.

Where shall we find a name worthy of higher admiration than that of the immortal Cuvier? and what biography can ever tell the extent of his indefatigable researches, and the number of his bright discoveries? Every thing bears the stamp of magnitude and greatness in his works.

Born at Montbeliard, on the 23rd of August, 1769, George Cuvier exhibited from his early years an extraordinary facility of conception, an aptitude for instruction. The most tender of mothers would often express her alarm for his health; for his complexion at this time indicated a delicacy of constitution, which premature study might probably enfeeble. She would rather have seen him fonder of his play than of his books: 'Le cœur d'un mere est le chef-d'œuvre de la nature.'

At four years of age he could read accurately; at fourteen he had finished his classical studies. But he was not satisfied merely with a knowledge of Greek and Latin; he paid much attention to history, geography, arithmetic, geometry, and drawing. Before he was fifteen years of age he had read Buffon through, commenting upon it as he went along, and copying the engravings of the different animals. By the patronage of Duke Charles of Wurtemburg, he was sent to prosecute his studies at Stutgard.* Some time afterwards he entered the establishment of the Count d'Hericy as tutor of his children. His cheateau of Pinquinville is situated not far from the sea-coast in Lower Normandy. Having a good deal of leisure time to himself, Cuvier devoted his mind to the pursuit of his favourite study, natural history. His fine talent for drawing enabled him to collect a large catalogue of the figures of all sorts of animals.

Appointed Professor of Natural History to the Central Schools, he obtained, soon afterwards, through the influence of M. Geoffroy St. Hilaire, the chair of Comparative Anatomy at the Jardin des Plantes in Paris.† While not more than 26 years of age, he was associated in the first organization of the Institute as a member of the physical and mathematical class.

During 32 years, Cuvier filled the place of perpetual secretary to the Academy of Sciences, with, we need

---

which probably indicates some inferiority in point of profound feeling. * * * Lord Byron's head is, I think, still more complete all throughout than that of Mr. Scott. The forehead is defective in much that Scott's possesses; but it is very fine upwards, and the top of the head is wonderfully capacious. Altogether, it is the finest in our time. I think it is better, on the whole, than either Napoleon's, or Goethe's, or Canova's, or Wordsworth's."

* His Diarium Zoologicum Primum bears the date of Stutgard.

† How long the French have been in advance of us in the noble study of comparative anatomy, appears from the circumstance of its having been recognized, as one of the departments of instruction, for at least 30 years before it was taught in this country. Fortunately, we have one mighty name to oppose to their phalanx of strength. Need we say that it is that of John Hunter?

scarcely say, the highest honour to himself and to his nation.

The duties of these numerous avocations would have been more than enough for most men — to him they were but as mere relaxation. Cuvier, whose name is worthy to be associated with that of Napoleon, and is one of the brightest ornaments of this or any other age, was not less distinguished in his administrative than in his scientific career. Every moment of his life was profitably employed: he was never idle. Even while sitting in his carriage, he was either reading or writing, or arranging some materials of thought. No author has written so many original works, with so little time devoted to them. Whenever he had a spare quarter of an hour, whether before dinner, or when attending a council of state or a sitting of the Institute, his mind was sure to be engaged in resuming some inquiry, which had been interrupted on the preceding day.

In the course of 1832, he experienced a degree of numbness in his right arm. This alarming symptom did not at all abate the energy of his mind. It was, alas! however, the precursor of a more complete paralytic attack, which proved fatal on the 13th of May.* He died with all that calm and dignified resignation which religion alone can bestow.

Our readers will probably recollect that, on dissection, the weight of Cuvier's brain was three pounds ten ounces and four drachms and a half— one-third heavier than an adult brain of ordinary dimensions." — *Caractères Phrenologiques.*

[*Remark.*—Much of the preceding eloge is very vague and inflated. We have, however, preferred to give it entire from the original, as a sample, among others, of the style of thought as well as of language, which French writers are so apt to indulge in.—*Rev.*]

* It is a singular coincidence that Napoleon, Sir Walter Scott, Canning, Wellington, Sir James Macintosh, and Chateaubriand were all born in the same year as Cuvier; and that Sir Walter Scott and Goëthe died during the same year as he did.

### Notice of the New French Phrenological Journal. M. Broussais on Education, &c.

Within the last twelve months, a new journal, devoted to the investigation of phrenology, has been started in Paris. The name which has been given to it is 'La Phrenologie.' It is edited by two doctors, MM. Place and Berigny, and by an advocate of the Supreme Court, M. Florens. They have obtained the co-operation of all the leading phrenologists in the metropolis; as, for example, M. Broussais, a member of the Institute, and president of the Phrenological Society of Paris; M. Bouillaud, a vice-president of this society, and well known as an indefatigable physician of La Charité Hospital; and M. C. Broussais, also a vice-president, and one of the professors to the Faculty of Medicine, and of the medical school at the Val-de-Grace. Among the others, we observe the names of Dr. Fossati, Dr. Leroi of Versailles, Professor Dumoutier, and M. Landrin, a barrister.

We propose to give an abstract of some of the most valuable papers in the numbers of this Journal, from its commencement last Spring.

M. Broussais, the elder, has contributed a series of papers on the *Application of Phrenology to the Education of Man.* These, however, are by no means very interesting, being far too much occupied with vague generalities, and sadly deficient in perspicuity and precision. We shall, however, extract a few of the better passages.

The writer commences his disquisition by pointing out the great divisions of the mental powers or endowments into instincts, sentiments, and intellectual faculties. The first division, that of instincts, is common to man with the lower animals; and their organs are first developed in the order of physical evolution. The second division, that of sentiments, comprehends the loftier moral attributes of man which belong to him as a social and moral being, such as conscientiousness, hope, veneration, &c.; and, in the third division, are arranged all the powers or faculties of the intellect, by which men become acquainted with the events and phenomena of nature, through the medium either of perception or of reasoning.

These three great divisions or classes of the mental powers are, according to the doctrine of phrenology, correlative to, or correspondent with, the three lobes of each cerebral hemisphere, the posterior, the middle, and the anterior. The instinct of amativeness is referred to the cerebellum; and perhaps therefore we may find the true cause of the tardy development of this feeling in the circumstance of its special localization, apart in some degree from the organs of the other faculties.

During the first period of life, the human being acts from mere instinct, without knowing what he does. Soon, however, some of the intellectual powers begin to manifest themselves; and, at the same time, some of the sentiments also are developed. These sentiments add their impulses to those of the instincts; and the intellect at this period of life has no other function than to shew to the one and to the other the external objects, which are to engage them. The intellect therefore in the young child is the handmaid, perhaps we should rather say the slave, of the instinctive and sentimental impulses. A change is soon to take place. The intellect, as it becomes more and more developed, acquires gradually an ascendancy over the other powers, instinctive as well as sentimental; and its various acts, or, more correctly speaking, the various phenomena of its action, become, as it were, so many "besoins." When this important change has taken place in the development of the mind, the person becomes a more reasoning, and therefore a more responsible, being. His actions are, or at least ought to be, no longer mere impulses of feelings; they should be the results of judgment and consideration. "Hence," says M. Broussais, "the whole problem of education resolves itself into merely hastening the time when the higher faculties will become, *if possible,* 'les besoins predominants, et les directeurs supremes' of the conduct of social man."

M. Broussais insists particularly on the qualifying expression "*if possible,*" in this proposition; as it is quite necessary to ascertain, in the first case, if there is in the child a sufficient amount of intellectual faculties, to lead us to expect that the exercise of them may become "le besoin predominant."

To determine this point, we should examine carefully the actual and the relative size of the frontal region. If it is fairly developed, we have good reason to expect success in our educational efforts; but if it is much depressed, and decidedly defective, compared with the size of the middle and posterior portions of the head, the discipline of instruction may do a good deal to improve the intellect; but we should most assuredly never anticipate a full extent of its powers.

This, therefore, is the first step which we should take in determining the capabilities of a child—viz. to ascertain the actual and relative size of the anterior lobes of the cerebral hemispheres. The next step consists in ascertaining whether the inferior or supra-ciliary, or the superior or frontal portion of the anterior lobes is most developed; in other words, whether the perceptive or the reasoning faculties of the mind are naturally predominant.

Again, in some children we find a remarkable quickness of apprehension on almost every subject; and others are distinguished by great natural acumen in one pursuit only, while they are dull and slow in all others. Thus one shews a remarkable talent for music; a second for drawing; a third for constructing figures; and so forth.

In some children, all the perceptive faculties are dull and inapt to apprehend, while the reflective and reasoning powers of the mind are vigorous and powerful. Now no education will ever force such children to be quick, smart, and brilliant in any pursuit. They may be compelled to study all the embellishing accomplishments of modern refined life; they may have masters and professors without end; see every thing; hear every thing; and taste every thing;—but all this will never make the youth a Sir Philip Sydney, or a second Crichton. And yet perhaps this very youth, who may perhaps have disappointed the fond hopes of an inconsiderate parent, will turn out one of the most valuable members of society. Although not bright and brilliant in fancy, not voluble and eloquent in speech, not sparkling with wit, or quick at gay repartee, and although he may have none of the attractive elegancies of high-bred education, or the graces of a refined sensibility, he may nevertheless be the delight and the boast of all who know him. It is precisely in such a character that we generally meet with that evenness of character, that clear-sightedness of judgment, that discriminative and fair and just balancing of events, and that intrepidity of resolution, which fit their possessor to steer his course "calmly and proudly" among the shoals and quicksands of life, whether, in the sunny hour of prosperity, or in the dark tempest of misfortune.

But to return to our subject. We have already said that, whenever there is a decided deficiency of development of the anterior cerebral lobes, we should never expect any great improvement of the intellectual powers from education. If the forehead, therefore, is much depressed, if the supra-ciliary portion also is very low, and not at all prominent, and if the distance between the external ear and the convexity of the frontal bone be decidedly very small, "jamais vous ne pouvez reussir à faire que les phenomenes intellectuels devinssent les besoins predominants d'un pareil sujet."

Again, if the superior transverse half of the forehead be broad and full, while the inferior or supra-ciliary half is flat or depressed, the individual will be found to be thoughtful and reasoning, but deficient in acuteness of observation, and in the memory of objects in general.

He will be satisfied with a few facts to ground lengthened commentaries upon, and he will be apt to be misled in the mazes of his own device.

Extreme cases of this description are not very unfrequently met with among idiots. Their want of the knowledge of facts seems to permit their minds to be entangled among the meshes of a misdirected and extravagant fancy; and thus such persons will be found to be continually framing some wild and aimless project, or indulging in some dreamy improbabilities. It is obvious that all attempts to reason them out of their reveries must fail, in consequence of the perception and memory being in them void, and, as it were, sieve-like, incapable of holding the images presented to their notice.

This conformation of cerebrum, and this variety of idiotism, is of rare occurrence, it must be acknowledged. In by far the largest proportion of cases, the whole extent of the anterior lobes, the reflective as well as the perceptive regions, is simultaneously atrophied.

Whenever the development of the two frontal regions is within the normal limits—the conformation of the immense majority of mankind—the intellectual powers may be brought by education to acquire the predominance in the mind; but at the same time we must remember that this predominance will always be apt to be disputed by the instincts and sentiments. If, however, this education is neglected, and if the mere wishes and desires of a child are invariably and quickly gratified, the reasoning powers, however well developed and however much cultivated in after-life, will never acquire that controlling influence over the actions of life, which we wish to see in every well-regulated mind. It is, therefore, of infinite importance to appeal frequently to the judgment and to the higher moral sentiments of a child, and thus to strive to strengthen and improve them, before the mere instinctive feelings of our nature have acquired from unchecked indulgence an undue degree of power. Should this discipline be neglected, "l'intelligence sera toujours l'esclave des instincts et des sentiments."

---

### Phrenology does not sanction a Belief in Fatalism.

The author of this article is M. Florens, an advocate practising at the Palais de Justice. He proposes to give a series of papers, illustrative of the beneficial application of phrenological discoveries to the science of legislation and jurisprudence.

"Before," says he, "entering upon the examination of the numerous important questions which arise from the application of phrenology to legislation, to the penitentiary system, and to the different modes of education at present pursued; before attempting to shew how this science — which constitutes the true philosophy of man—founded, as it is, on his organization, and on a rigid examination of the phenomena which it produces, must assume a high place among the various branches of the social system; we deem it necessary to examine somewhat minutely an objection, which our opponents have fondly been in the habit of advancing, ever since Gall first propounded his discoveries. It has been said that phrenology leads directly by its doctrines to fatalism, and that, by sapping the foundations of the moral code, it palliates iniquity and crime.

If phrenology, it has been argued, be true, teaching as it does that all the faculties of the mind, all the propensities of our nature have their seat in the brain, that the development of these faculties and propensities is the law of their nature, it must follow that man is irresistibly urged on to virtue or to vice, that he has no choice in his actions, and therefore that he cannot be deemed responsible for them. Again, as phrenology asserts that the development of an organ may be recognized by a protuberance on the outside of the cranium, our opponents have improved upon the assertion, and made us say, that "a man, in virtue of his organization, possessing the bump of theft, or that of murder, obeys the dictates of his nature when he steals or kills; he has no freedom of will; and consequently he cannot be properly accounted to be responsible for his actions."

It is truly astonishing what credence this most erroneous argument has obtained. From the gossip of the parlour it has reached the disquisitions of the lecture-room, and even the more solemn deliberations of our halls of justice.

"I have often," says M. Florens, "heard it said at the *Palais*, 'Phrenology is a dangerous science; it proclaims that there is in human nature a disposition to murder, to steal, &c.; that we are irresistibly, and, as it were, fatally, urged on to the commission of vice. And, if such principles be just, how can any punishment be ever inflicted upon a criminal? With the system of the phrenologists, neither morality, religion, nor legislation can possibly exist.'"

If phrenology indeed taught such doctrines, well might our opponents call it a most dangerous science. They have conjured up phantoms of their own making, and most unjustly attribute them to us. All that phrenology has ever asserted in regard to the functions of the brain is, that there is a multiplicity of organs belonging to it, and that each organ has a distinct function. It has shewn us the admirable provision of Nature in always placing the antidote or counteracting agent at the side of the evil propensity, and it has proved to us, beyond all doubt, that a wonderful harmony exists between the machinery of the human mind and the plan of universal creation. In no department of physical nature do we perceive unmingled happiness, or the existence of any one good or any one enjoyment, without a corresponding evil and distress. The helpless inoffensiveness of the lamb does not save it from the gripe of the wolf or of the eagle; the timid mouse falls a prey to its cunning enemy; and how continually are the gay tenants of the wood sacrificed in wanton sport by man himself.

Every thing shews a checquered state of joy and misery, of happiness and pain, of good and of apparent evil; and if such be true of all the other works of creative power, why should we expect that the human mind should be excepted from the law?

The more we examine the physiology of the mind, the more completely shall we see the truth of those great principles which Gall and his disciples have so incontrovertibly established, that there is a multitude of innate propensities, feelings, and faculties, and that many of these, if unchecked and uncontrolled, lead on the individual to crime and to misery. If this proposition be true, and we most firmly believe that it cannot be gainsayed, then phrenology, instead of encouraging vice or palliating crime, must be regarded as by far the purest system of ethical instruction, inasmuch as it discloses to our notice all those wayward and misleading impulses of the mind, from which wickedness and its attendant suffering arise.

By pointing out the various faculties and endowments of each individual, phrenology affords him the means by which, under proper guidance, he may check some, encourage others, and regulate and direct them all.

But it may be said that phrenology asserts that, in certain persons, the evil propensities are so predominant, in consequence of their inordinate development, or of the imperfect growth of the higher sentiments and faculties, that we cannot fairly regard them as responsible for their actions.

This is an exaggerated view of what has been laid down in phrenological works. All that has been insisted upon is simply this—that there is a great difference in the criminality of different persons culpable of the same offence, and therefore that, as all are not equally responsible, all should not be punished alike. In those in whom the lower propensities are active and powerful, and in whom perhaps at the same time there is a deficiency of the higher sentiments and of the reasoning powers, the impulse must be, as a matter of course, much stronger than in those who are more favourably organized; and consequently the crime cannot be regarded so heinous, in a moral point of view. The law itself, founded though it be on the mere maxims of political expediency, pays some consideration to certain extenuating or palliative circumstances in each criminal's case.

Even in the most unfortunate examples, where the tendency to some particular vice is very predominant, the phrenological code is far from being indiscriminately lenient.

The evil propensities never exist alone, or unassociated with a certain development of the virtuous feelings of our nature; and, if these be uniformly neglected, and if no efforts have ever been made to cherish their growth and to control the evil passions, the individual cannot reasonably hope for leniency from offended Justice.

We see, therefore, that phrenology is very far from inculcating the pernicious doctrines of a blind fatalism, and of unavoidable sinfulness and crime. Almost every human being is a free-acting and responsible creature, and consequently deserves punishment when he infringes the laws — the punishment being, as far as possible, proportionate to the development of his cerebral organization, and to the circumstances of each individual case. The great aim and object of every wise and merciful legislation should ever be, to prevent, as far as possible, delinquencies and crimes, to punish the wilfully guilty, and to protect society from those who are incorrigible, by a regulated system of confinement and discipline. — *La Phrenologie.*

[*Remarks.*—There is much good reasoning, and some vivid illustration, in the preceding observations. Perhaps the chief error of M. Florens lies in the ambiguity, or, we should rather say, the incorrectness of his language. He talks of originally good and evil propensities, instinctive tendencies to virtue and vice, &c. Now, in truth, none of the propensities or feelings of our character are by nature primarily evil or vicious. It is the abuse or misdirection of them that renders them so. Amativeness and Combativeness, when uncontrolled by the judgment or by the moral sentiments, lead to crime and punishment; but, when obedient to the higher man, they become the source of some of the purest delights, and some of the noblest actions of our nature. So it is with the other propensities.—*Rev.*]

### Alleged (!) Affection of the Organs of Calculation.

M. P., a merchant tailor, and head of a very large establishment, had been occupied for upwards of a twelvemonth with minute and laborious arithmetical calculations, to which he had been little accustomed previously. He began to experience a sense of weight and uneasiness in the lower and lateral part of the left side of the forehead; and this unpleasant feeling was frequently followed by darting and tensive pains in the region, where are seated the organs designated by the numbers 28 and 29 in the phrenological table, those of Calculation and Order. The face was pale; the pulse was small and contracted; the appetite was perfectly good, and the bowels were open. He was leeched, bled to a small amount, and used sinapised foot-baths. Dr. Place, being called in, recommended repose, and half a grain of opium, in the form of a pill, at bed time. But this treatment did not produce any benefit. In spite of his anxious desire to recover his health, the patient could not prevent his mind recurring constantly to his book-keeping and accounts.

Dr. Place now endeavoured to subdue the excitement of the organs (calculation and order) affected, by exciting the action of some of the adjacent ones. For this purpose he suggested to the patient to indulge in light cheerful conversation, to avoid all serious and thoughtful discussions, and to select for reading certain "ouvrages gais futiles, dont je lui prescrivis en quelque sort la dose, c'est à dire un volume, puis deux, puis trois." The writings of Paul de Kock were particularly recommended to his notice. The piquant style of this gay and spiritual painter of manners is admirably calculated to dissipate the gloom of ennui, and in such cases as the present, is the most effectual as well as the most pleasing remedy which can be employed.

The object, which Dr. Place had in view, in recommending this style of reading to his patient, was to excite the activity of some of the intellectual faculties, which had for a length of time been allowed to be very inactive. In acting upon them in a *revulsive* manner, the two suffering organs were brought to a state of complete repose, while those of ideality, mirth, eventuality, colour, &c., were agreeably and constantly occupied. As an adjuvant, Dr. Place recommended the use of *café à l'eau* as a general stimulus to the whole cerebral mass. It succeeded admirably well. The patient found his spirits gratefully excited, and his sleep became more refreshing. The coffee, in conjunction with the "therapeutique phrenologique" indicated above, seemed to restore a general equilibrium of the cerebral organs.—*La Phrenologie.*

[*Remarks.*—We leave our readers to make their own comments on the preceding details. A century of similar cases, minutely examined and carefully reported, may possibly throw some light on phrenological pathology. At present, all is uncertain and very obscure.]

---

### Apoplexy—Loss of the Memory of Words.

M. V., sixty-one years of age, exhibiting all the appearances of the sanguineous temperament and obliged by the nature of his occupations to live a sedentary life, had enjoyed good health up to the period of the first attack, twelve years ago. He was seized with a general feebleness, and slight numbness of the entire right side, more particularly of the right arm. The use of local and general bleeding, of a seton in the back of the neck, and of purgatives internally, removed completely every unpleasant symptom, and he continued to enjoy moderately good health for the next twelve years. In November, 1836, after rather laborious exercise, he was siezed, while sitting at table, with an impediment of speech, and with powerlessness and diminished sensibility of the right side. Leeches were applied to the anus, and on the following day he was bled from the arm. For several months he remained in the same condition, with all his intellectual faculties quite clear, but not able to express his thoughts or feelings in words. Of late he has acquired a partial degree of facility in pronouncing certain words. At present he is very irritable; the right side is somewhat more feeble and less sensible than the left one. He complains occasionally of headache, which he refers to the occiput and sinciput. He retains all his mental faculties nearly quite entire, and exhibits no mental decrepitude, save the loss of the memory of words; for the memory of places and that of facts remain unaffected.

The preceding case may be adduced to shew that the different parts of the brain act, in many cases at least, independently of each other, and that the functions which they exercise may be perverted, or altogether destroyed, without being necessarily attended with any affection of the entire organ. We must acknowledge indeed that it is of rare occurrence that one faculty or power only of the mind is disturbed, while the rest retain their normal integrity; and this, be it remembered, is altogether in accordance with what we might have anticipated from pathological anatomy, seeing that any lesion of the cerebral texture is seldom limited to one small spot only. M. Rochoux indeed has, in the last edition of the Dictionnaire de Medecine, asserted that, in the present state of medical science, there are not any sufficiently authentic data to warrant us in saying, that we can distinguish the lesions of one part of the brain from those of any other part. While we are willing to admit the difficulty of the subject, we should remember that there are the high authorities of Foville, Serres, Bouillaud, and Pinel Grandchamp, to oppose to the statements of M. Rochoux.

From the ample mass of facts collected in many years experience, M. Foville has come to the following conclusions relative to the symptomatology of cerebral disease: that, when there is a lesion of the optic thalami and their radiations, the upper extremities are usually more or less affected; that, when it exists in the corpora striata and their radiations, the lower extremities usually suffer; that the grey or cortical substance of the convolutions is the seat of the intellectual functions, and that the fibrous substance and the central parts are the seat of the voluntary movements.

To recur, for one moment, to the subject of the case which we have reported above, it is worthy of notice that the loss of speech may depend on two very different causes; either a paralysis of the muscles which move the tongue, or on a forgetfulness of the words which serve to express our feelings and ideas. In the subject of the present remark, the tongue retained all its varied movements in every respect; there was no appearance of paralysis or loss of power either of the lingual or of the buccal and palatal muscles, and mastication was performed without difficulty. The seat, therefore, of the affection, which had induced the loss of speech, must have been in some other part; and in all probability it was in that portion of the brain which phrenologists have assigned as the organ of language.—*Ph. Gouly.*

---

DELIRIUM —ALLEGED AFFECTION OF CERTAIN ORGANS.

A restaurateur at Versailles, 27 years of age, and of a highly sanguineous temperament, was suddenly seized, in the summer of 1833, with severe headache and loss of consciousness, after having been for some time exposed to the direct rays of the sun. On the following day, he became occasionally delirious, and, in the intervals of sanity, he referred the pain to his temples almost immediately above the ears. The heat of these parts to the hand was greater than that of the rest of the head. The delirium consisted in an excessive dread of robbers and assassins; he believed that there were persons constantly in his room, opening his desk, and threatening to murder him. He would get out of bed, try to make his escape, and threatened to kill any one who came near him. He was bled freely on two successive days; but the pain in the temples and a certain degree of the peculiar delirium still remained. Twenty leeches were put on each side of the head, and ice was ordered to be kept constantly applied afterwards. The symptoms now quickly subsided; and the patient was soon able to resume his business.

During the following Winter, this man was again seized with symptoms of high cerebral excitement. The pain, however this time, was chiefly in the anterior and lower part of the forehead and between the orbits, and the character of his delirious delusions was very different from what it had been during the former attack. There was now no dread of robbers and assassins about him. Instead of these unpleasant neighbours, his mind's eye perceived animals of all sorts, fruits, articles of furniture, which he would describe minutely to the attendants, and wish to be brought to him. A number of leeches was applied over the seat of the pain, and the use of the ice was resumed.

Since the date of this second attack, the patient had several relapses; and in April, 1836, he died suddenly, while in bed. It was in all probability an apoplectic seizure; but, as there was not a dissection, the exact nature of the fatal attack is necessarily somewhat uncertain.

The reporter of this case, Dr. Leroi, in his remarks on the peculiar character of the delusions in the two attacks, of which we have given a short description, calls the attention of the phrenological reader to the accordance between them and the normal functions of the organs affected.

In the first attack, the seat of the pain was immediately above and around the ears, and the delusions all bore reference to the dread of being plundered or killed, and to the means of defending himself. Now the phrenological organs, which we may suppose were chiefly affected, are those of *the instinct of self-preservation, the instinct of destruction, the sentiment of property,* and lastly *cunning*, according to the nomenclature of Gall; or of *destructiveness, combativeness, secretiveness,* and *love of life,* according to that of Spurzheim. How rational, therefore, is the interpretation of the existing delirium, according to the principles of phrenology! Thus the very aberrations of the mind very beautifully illustrate the doctrines of these two great mental physiologists.

The reader cannot fail to have remarked the very striking difference in the mental illusions during the two attacks, to which we have alluded in the preceding report. During the first, the mind seemed to be completely and solely occupied with ideas of self-preservation and defence; whereas, during the second, when the local pain indicated the lower part of the anterior lobes to be chiefly affected, the images were those of various objects, as different kinds of fruits, animals, and articles of furniture, which the patient would in his fancy take hold of, and distribute to those around him. Now the phrenological organs, which we may fairly believe were then most affected, were those of Form, Size, and Individuality.—*La Phrenologie.*

*Remarks.*—We fear that all this is much more ingenious than probable. In giving this and the other cases from the reports of the French pathologists, we are very far from yielding our assent to their reasoning. To us it seems very fanciful and conjectural.—*Rev.*

---

### Demonomania, with Delirium.

Allais was an honest workman at Versailles, well known to his neighbours as a good husband and kind father. His temper, however, was always rather testy and irritable; he could not bear a joke upon himself or any thing connected with him; and his "amour propre" was thus exposed to very frequent offence. One day his comrades had jestingly called him *Mouchard;* and from that time he seemed to be affected with a malaise and disquietude, which haunted him continually. On the 29th of February, when his doctor went to visit him, Allais opened the door, with no other covering upon him than his shirt. What was the astonishment of the doctor on entering the room, when he saw this man's cat and dog, "animaux qu'il aimait," lying strangled on the floor. Upon asking him the cause of this strange violence, his only answer was, "Il le falloit." But the surprize was soon converted into horror, when he found in another room Allais' poor wife stretched out lifeless on the bed. The madman had strangled her also. When reproached with the deed, he coolly replied, "It was quite necessary, for she was the devil who wished to kill me;" and then set about repeating the litany of the Holy Virgin.

Allais was, in short, labouring under that form of insanity, which has been denominated *demonomania*. By his answers to various questions, it was discovered that during the night of the 28th, he awoke half-suffocated, suffering from a dreadful constriction of the chest, which he thought was caused by the devil. His next fancy was that the devil had assumed the shape of his wife; and under this wild delusion, he had committed the dreadful act. When he had killed her, he then strangled his dog and cat, always believing that the devil had passed into their bodies.

When taken to the "maison d'arrêt" at Versailles, Dr. Leroi examined very carefully his phrenological development. The forehead was narrow, and flattened on the temples; the head rose high to the vertex, and was especially full round the ears. The organs of veneration and of marvellousness were rather largely developed; and likewise those of destructiveness, self-esteem, and love of approbation. The organs of almost all the other sentiments were small, and those of the intellectual faculties were at the *minimum* of development.

Such is the organization of this man—an organization, which in our opinion affords an indubitable example of the truth of phrenology. (!)

Wounded in his *amour propre* and his vanity by the foolish gibes of his associates, he became fretful and irritable; his brain gradually began to suffer, and at length insanity followed. Now what was the character of this insanity? He is haunted with visions of the devil, and strives to get rid of him. Impressed with this delusion, he kills his wife, and then his dog and cat; and, after all, he sets himself to pray and to recite the litany. The active impulses to this train of actions were undoubtedly self-esteem, vanity, marvellousness, veneration, and destructiveness. These various feelings were unchecked and uncontrolled by the intellectual faculties, which, we have mentioned above, were very imperfectly developed in his character.

If, after these details, we now ask ourselves what treatment should be followed in such a case as the preceding, phrenology answers that the only rational hope of doing any good must be founded in the attempt to excite some of the other feelings and faculties of the mind, which have hitherto been dormant and unemployed, and the exercise of which may counteract the morbid excitement of the offending ones.

This is the only true and philosophical method of treating most cases of insanity; viz. by trying to substitute healthy for deranged action. We daily see the necessity for such a course in those eccentric individuals, in whom a single idea is apt to usurp a dominant influence over the mind, and who would certainly become decidedly monomaniacal, if no repose was given to the over-excited organs, by calling into play other organs which have been long in a state of inaction.

Gall mentions that in his own case he was frequently obliged to abandon for a time the investigation of a particular idea, which had long occupied his mind, and had so excited it as to bring him to a state approaching to derangement. "I was forced," says he, "to create for myself a new and favorite occupation. I betook myself most zealously to gardening, and at length succeeded in this way to re-establish the equilibrium of my intellectual powers."

We must ever remember that to effect this important object—the tranquillizing of the morbidly-excited feelings by the substitution of those which have been feebly exercised—it is necessary that there is no "vice d'organisation" of the brain; in other words, that there is not an inordinate development of one part, and an imperfect development of another.

If such irregularity or disproportion does exist, we cannot fairly hope for much success in our curative efforts.

Unfortunately, this is the case with poor Allais, the subject of the preceding report. In him the intellectual faculties are disproportionately feeble and "impuissants," while some of the sentiments and feelings are inordinately strong. However humiliating such a reflection may be, and however unwilling we are at first to admit that some human beings are so organized that they can have little control over their impulses and emotions, we must be on our guard not wilfully to shut our eyes against the truth. Let us not forget that a doctrine, not dissimilar in its principles, is inculcated in the holy writings, where the Apostle of the Gentiles hath said, "For the children being not yet born, neither having done any good, or evil, that the purpose of God according to election might stand, not of works, but of him that calleth;" and again, "Therefore hath he mercy on whom he will have mercy, and whom he will he hardeneth."

It is difficult indeed for the finite mind of man to reconcile such a doctrine with our notions of impartial justice. Instead, however, of attempting to solve the mystery, let us rather argue in the words of St. Paul, "Thou wilt say then unto me, Why doth God yet find fault? for who hath resisted his will?

Nay, but oh! man, who art thou that repliest against God? Shall the thing formed say to him that formed it, Why hast thou made me thus?

Hath not the potter power over the clay, of the same lump to make the vessel unto honour, and another unto dishonour."—*La Phrenologie.*

---

Account of Vito Mangiamele, the Youthful Prodigy of Calculation—the Phrenological Development of his Head, &c. &c.

The French Institute have, at some of their recent sittings, been a good deal occupied with examining the arithmetical and mathematical powers of this extraordinary child. The public newspapers have related many of the most striking proofs of his wonderful talents, as exhibited in the answers which he gave to numerous questions proposed to him by the academicians. As no one can call in question the correctness of the statements alluded to, we deem it unnecessary to reproduce them here, and shall therefore proceed to examine the phrenological development of Mangiamele's head, and to ascertain whether the objections derived from this examination are, as has been alleged, unfavourable to the doctrine of phrenology.

The child is ten years of age, of an ordinary stature, and of a sound constitution. Under the appearance of an almost apathetic tranquillity, there is a great cerebral activity, indicated by the vivacity of look, the expressive cast of features, and the promptitude of reply on all occasions. The forehead is full and largely developed; it rises almost perpendicularly from the root of the nose. It is remarkably prominent in the middle, in consequence of the unusual size of the organs of individuality, eventuality, comparison, and causality. On the outer side or boundary of the two last-named organs, the forehead is retreating (parait encore fuyant). This is attributable to the feeble development of the organs of gaiety, and some of the adjacent ones — an organization quite in accordance with the character of the child, who is much more remarkable for its thoughtfulness and reserve, than for the playful cheerfulness of its disposition. If we examine the distance between the meatus auditorius and the most projecting point of the os frontis, it will be observed that the anterior part of the head has a great depth, compared with the average depth of the part in young children.

The organs of eventuality and locality are very large, and, on the contrary, those of time and tune are relatively small — a development which gives a compressed and narrow aspect to the lower part of the forehead. The row of the perceptive organs along the arch of the eyebrow, and including those of individuality, form, size, weight, colour, order, and number, deserves especial notice. When the three first are feebly developed, the nasal prominence is large, the inner angles of the eyes are near to each other, and the root of the nose looks as if it had been pinched and compressed between the fingers. Such a conformation is observed in those persons who cannot readily perceive, or call to mind, resemblances in objects. On the contrary, in those who excel in quickly seizing and remembering forms, proportions, and features of objects—as in physiognomists, portrait-painters, and in all persons, who are in the habit of saying that they can see in their *mind's eye* things and beings as if they were before them—the root of the nose is almost always large and prominent, the arch of the eyebrow commences in an inclined plane from below upwards and from within outwards, as we observe in the portrait of Vandyke, and also in young Mangiamele, who has a remarkable talent for the memory of things: "il voit mentalement les objets ou les signes des pensées dans leur place et leur ordre respectif, comme s'ils etaient ecrits sur un tableau."

The organs, situated over the middle portion of the eyebrow, are not largely developed; the eyebrow, therefore, at this part is flat, and not bulging as we see it in the heads of famous painters, like Rubens, Rembrandt, and Tintoretto, in whom the centre of the eyebrow is lofty and prominent.

The outer portion of the eyebrow is remarkably full in the head of the young Mangiamele. A similar development has been observed in Colborn, Buxton, and other arithmetical geniuses, who have shown a striking talent for numerical calculation from their earliest years.

Mangiamele was only four years of age, when he first gave proofs of his remarkable aptitude for cypering. He detected some errors in a long account, which his father had been calculating. From that date up to the present time, his mind has acquired greater and greater facility at all arithmetical operations.

But to return to his cerebral developement, we are told that Nature has stamped on the forehead of this boy the seal of Pythagoras, Archimedes, Euclid, Newton, and Kepler. In him, as in them, the outer extremity of the eyebrow is elevated and prolonged backwards, the external orbitar angle of the frontal bone is depressed and advanced above the external angle of the eyelids, so that the forehead, at that point where the organ of number is situated, is remarkably prominent.

The extraordinary powers of this child are not limited merely to the faculty of remembering figures; for, besides this, he can work them, through some of the most complex operations of arithmetic, with surprising rapidity and correctness. Moreover, he boldly enters on algebraic speculations, and has taught himself to resolve equations even of the fifth degree. In working out many of these difficult problems, he pursues a method peculiar to himself, and which he has devised in his own mind.

The reporter, M. Dumoutier, says that, after continuing for some time calculating, the subject of the preceding remarks sometimes complains of a pulsatory pain on the outer part of the temples, almost exactly over the organ of number.

Appended to the account now given of this youthful prodigy of calculation, there were some observations of the elder M. Broussais, in answer to certain misstatements, in the public journals, relative to Mangiamele. It has been asserted that the organ of calculation was by no means very largely developed in his head. Now this assertion, says Broussais, is directly contrary to fact. The organ is full, since it maintains the elevation and the horizontal position of the extremity of the eyebrows at the temples. But in truth it is not necessary for the truth of phrenology that this organ should be enormously developed, seeing that the prodigies of calculation do not depend *solely* on the mere memory of numbers.

What is most astonishing in this child proceeds from the faculties of form, individuality, comparison, and causality.

In conclusion, it deserves to be noticed that the Minister of Public Instruction, impressed with the importance of justly educating such talents as those possessed by Mangiamele, has written to the Secretary of the Academy the following letter: "The Academy of Sciences has appointed a commission to examine the young Mangiamele. I request you to direct the attention of the Academy to the mode of education, which should be given for the purpose of insuring a quiet and regular development of his faculties. I shall do all in my power to facilitate this important object. France is the adoptive country of all talents."—*La Phrenologie.*

In a following number of the same journal, M. Florens, the advocate, resumes the subject of Mangiamele's organization. He invites all the anti-phrenologists to examine his head and satisfy themselves, whether the organs of number and causality are not very largely developed. At a lecture recently given by M. Place, the boy himself was present, and quite astonished many able arithmeticians by the extraordinary rapidity and correctness in solving the most complicated calculations.

---

### Organization of the Brain in the Negro compared with that in the European, and also in the Ourang-Outang, by Professor Tiedemann.

The disgraceful treatment of the negroes by all the civilized nations of Europe will ever reflect the greatest opprobium on humanity. Slavery indeed, it must be confessed, seems to be coeval with the earliest historic records. It existed among the Phœnicians, Jews, Egyptians, Greeks, Romans, and Saracens, and perhaps might have existed to the present day among ourselves, had not the beneficent spirit of Christianity softened and improved the mind of man.

The traffic in negro slavery by Europeans seems to have commenced about the middle of the fourteenth century.

One of the pretended motives, which the palliators of slavery have often brought forward in defence of this abominable traffic, has been the base and dastardly allegation that the negro race is altogether a degraded and inferior section of the human family—as if the Creator had thus, in some measure, intended that they should be made the slaves of the more highly-organized tribes. This unworthy doctrine has been, if not openly avowed, certainly not contradicted by naturalists of the greatest eminence, such as Camper, Soemerring, Cuvier, Lawrence, &c., all of whom have either expressly asserted, or allowed their readers to infer, that the negroes were inferior to the white races of mankind in the organization of their cerebrum, and therefore in their intelligence and capacity for mental improvement.

The great object of M. Tiedemann's recent inquiries has been to ascertain if there be any truth in such opinions. For this purpose, he minutely examines the following two questions:

Is there any essential and important difference in the gradual development of the cerebrum in the negro from that in the European? and, secondly, is there a greater resemblance in the brain of the negro than in that of the European, to the same part in the ourang-outang?

*Weight of the Brain in the European.* M. Tiedemann, having carefully weighed the brains of 35 men and 17 women, has drawn the following conclusions from his experiments.

1. The weight of the brain in an adult male European varies from 3 lbs. 2 oz. to 4 lbs. 6 oz. (the pound consisting of 12 oz.)

The brain in men, distinguished by their intellectual endowments, is usually very large. That of the celebrated Cuvier weighed 5 lbs. and between 3 and 4 oz.;* and in the late Baron Dupuytren it weighed 5 lbs. and 4 oz.

---

* This account appears to be strangely at variance with what has been stated

On the contrary, the brain in persons of small intellectual capacity is generally small; and in congenital imbeciles or idiots it is still more strikingly so. The brain of an idiot examined by Tiedemann was found to weigh only 1 lb. 8½ oz.; and in another it was 1 lb. 11 oz.

2. The brain in the female is rather lighter than in the male. The usual weight may be stated to vary from 2 lbs. 8 oz. to 3 lbs. 11 oz. Tiedemann has never yet met with a female brain which weighed 4 lbs. In a female idiot it weighed only 1 lb. 6 oz.

On the whole, it may be asserted with truth that the brain in the female usually weighs from 4 to 8 oz. less than in the male. The brain of the new-born female infant weighs less than that of the male infant.

3. The brain does not attain its full development until the seventh or eighth year of life. Soemerring is, therefore, quite mistaken in asserting that the brain does not increase in dimensions after the end of the third year.

Drs. Gall and Spurzheim supposed that this organ continues to increase gradually till the fourteenth year. The brothers Wenzel have, however, shewn that it reaches its full development about the eighth year; and this opinion is confirmed by the remarks of Hamilton.

4. Desmoulins is of opinion that the volume of the brain actually diminishes in old age. He attributes the decay of the animal and intellectual energies to this cause.

The Wenzels and Hamilton, on the other hand, have denied the truth of the assertion.

It is, however, worthy of remark, says Tiedemann, that the brain of an old man whom I examined, 82 years of age, was very small, and weighed only 3 lbs. 2 oz.

In general, too, I have found that the cavity of the cranium is certainly of a somewhat smaller volume in old than in middle age: it seems therefore probable that the brain does actually diminish after a certain period of life.

5. It is very evident that there is a strict connexion between the absolute volume of the encephalon and the power and extent of the mental faculties.* We have already alluded to the unusual size of the organ in those of high intellectual capacities, and to its shrunk size in idiots and imbeciles. Many other observers, besides Drs. Gall and Spurzheim, have much insisted upon these points.

The weight of the encephalon generally remains the same, although the other parts of the body may become either greatly increased or diminished in their dimensions. Hence the brain is relatively larger in lean than in lusty persons.

At the period of birth the relative size of the brain is very large, weighing nearly one-sixth part of the whole body. It becomes gradually less and less as the child advances in age. Thus at the end of the second year, the relative bulk of the brain to that of the whole body is as 1 to 14; at the end of the third year, as 1 to 18; at the end of the fifth year, as 1 to 24; and at a subsequent period, it is not more than as 1 to 35. This last proportion continues, with a slight change, during the rest of life; varying, as a matter of course, very much according as the person is lean or very stout.

The brain of the female is very often somewhat larger than that of the male, in proportion to the bulk of the whole body.

---

a few pages back, in the description of Cuvier's character. The weight of his brain is there mentioned to have been 3 lbs. and rather more than 10 oz. There must be a diversity in the weights employed. Perhaps one was by the German and the other by the French scale.

* Such an admission as this from so distinguished an observer cannot fail to be acceptable to the phrenologist. Be it remembered, however, that, in estimating the power and extent of any mind, attention should always be paid to the temperament of the individual.

*The Brain of the Negro.*—The brain of a young negro, 14 years old, was found by Soemerring to weigh 3 lbs. and rather more than 6 oz. Sir Astley Cooper weighed the brain of a full-grown negro, and found it to be only 3 lbs. and 1 oz. The brain of a negro, who died at Liege, weighed not more than 2 lbs. and 3 oz.

Such isolated statements as these, however, cannot go for much in assisting our present inquiries. It would be necessary to weigh the brains in a number of negroes about the same time of life, before we can hope to attain to any accuracy on the subject; and hitherto we are not provided with data sufficiently numerous and accurate. We are, therefore, obliged to have recourse to the measurement of the capacity of the cranium to assist us to determine the point.

*Capacity of the Cavity of the Cranium.*—The crania, in M. Tiedemann's experiments, were first weighed with, and also without, their inferior maxillæ: they were then filled with millet seed by the occipital foramen. The increase of weight gave their volume or capacity. By examining in this manner 41 crania of the negro race, and 77 crania of the Caucasian race, he arrived at the conclusion that the capacity or volume of the cranium in the former is not at all smaller than in the latter.

M. Hamilton has confirmed the accuracy of this statement by his own researches.

These facts, says M. Tiedemann, demonstrate that the opinion of Camper, Soemerring, Cuvier, Lawrence, Virey, &c., as to the inferior development of the negro cranium, is quite at variance with the truth. The first of these naturalists was led into error by taking what he called the facial angle as the guide of his measurements. According to the researches of M. Tiedemann, such a measurement is extremely apt to be fallacious in estimating the absolute size of the cerebrum. So much for the encephalon in negroes.

M. Tiedemann then compares the medulla oblongata and spinalis in the negro with the same parts in the European; and he assures us that in no respect is there any difference appreciable. Soemerring had asserted that some of the cerebral nerves, especially the olfactory pair, are smaller in the negro than in the white race. This too is quite a mistake, according to Tiedemann.

With respect to the brain in the ourang-outang, when compared with that in any of the races of mankind, M. Tiedemann states:

1. That it is smaller, lighter, and more contracted in all its dimensions, as from above below, from forehead to occiput, and also from side to side.

2. That, in proportion to the size of the nerves which issue from it, it is smaller than the human brain.

3. The cerebral hemispheres, relatively to the medulla spinalis, the cerebellum, the corpora quadrigemina, and the thalami optici, are smaller than in the human brain.

4. The convolutions and the furrows are not so numerous or so deep.

5. The only resemblance which can be seen in the brain of the negro and that of the ourang-outang, consists in the convolutions and furrows being more symmetrical than is observed to be the case in the brain of the European.

From a review of all his researches, M. Tiedemann has drawn the following conclusions respecting the brain in the negro.

A. That the brain in the negro is, in its totality, as voluminous as in the European, or in any other of the human races. The weight of the organ and the dimensions and capacity of the cranium demonstrate the truth of this statement.

B. The cerebral nerves in the negro are, relatively to the size of the encephalon, neither larger nor thicker than in the European. The assertion, therefore, of Soemerring and his disciples to the contrary is quite erroneous.

C. The outer surface of the cerebellum and of the medulla oblongata and medulla spinalis in the negro, is altogether similar to that in the European.

D. The internal structure, and the distribution of the cortical and medullary substance exhibit no traces of any peculiarity in the negro.

E. The brain in the negro differs in no respect from that in the European, except *perhaps* that its convolutions and grooves appear to be "un peu plus symmetriques:"—even this slight peculiarity is probably not constant.

M. Tiedemann, having thus discussed the organization or development of the brain, then proceeds to offer a few remarks on the intellectual capacities of the negro.

As the brain is the organ through the medium of which we exercise the powers of mind, in the same way as the eye is the organ of vision and the ear of hearing, it is but reasonable to suppose that the extent and activity of these powers may vary with the development of the organ. Admitting the truth of this position, it has been rashly asserted by some that the negro race *must be* necessarily inferior to the rest of mankind, in consequence, it has been said, of the lower degree of their cerebral organization.

Our author has ably shewn the utter groundlessness of the premises, and he has thus most triumphantly overthrown the odious doctrine which has been founded upon them. He has zealously endeavoured to prove that the mental capacity in the negro is by nature as great as in any of the white races; and closes his ingenious disquisition by appealing to all the best feelings in favour of the long-despised and oppressed people of Africa.—*Gazette Medicale.*

---

ON ENCEPHALIC IRRITATION—ARACHNITIS, HYDROCEPHALUS ACUTUS, &c.—IN INFANTS, BY M. PIORRY. TREATMENT OF CERTAIN CASES BY ENEMATA OF PERUVIAN BARK.

*(A Memoir read before the Royal Academy of Medicine.)*

The knowledge of diseases, essentially founded on the anatomy and physiology of man in health and in disease, has made in our days indisputable progress. We have already discovered, in a series of determinate symptoms, the primitive source of the general phenomena which are manifested. We have seen that excitation, irritation, congestion, phlogosis—limited usually to one organ, sometimes to one apparatus, more rarely to one system, and least frequently still to several tissues viewed as a whole—are the local causes of constitutional disturbances. Such an improved etiology has necessarily led to an improved method of treating diseases. We must confess, however, that the success of our treatment has by no means corresponded with our advances in pathological anatomy; and certainly in no disease does this remark hold so true as in the malady, to which we have given the name of Infantile encephalic irritation, and which has been by different writers called Hydrocephalus acutus, cerebral Fever of infants, Arachnitis, &c. &c. This very diversity of nomenclature is a sufficient proof that the knowledge of this disease is still very imperfect.

Some writers regard it as an essential febrile affection, usually of an ataxic or malignant type; and hence they have denominated it as infantile cerebral fever, a name which M. Gendrin has of late years wished to restore. Cullen, as is well known, classed it among his apoplexies; and Pinel treated of it under the head of dropsies, and retained the name of *hydrocephale aigue.*

The admirable pathological researches of MM. Lallemand, Rostan, and Martinet have shewn how very often it is connected with an inflammation of the arachnoid membrane. M. Piorry, however, confesses that he is far from being satisfied with the doctrine of limiting the disease to a morbid state of the arachnoid membrane alone. He remarks, very justly, that the arachnoid membrane does not seem to exercise much influence on the cerebral functions in a state of health, and that it can be, only by its contact with the brain, that it can affect these functions. If arachnitis is accompanied with delirium, this symptom must be attributed only to the sympathetic, perhaps coexistent, irritation of the cerebral substance; if it is attended with spasmodic contractions, disturbance of the sensual perceptions, &c., the real seat of these morbid phenomena is no doubt in the encephalon itself. The old hypothesis of sensibility being in any way resident in the cerebral membranes is now very justly exploded.

We are thus almost forced to the conclusion that the symptoms of the disease, which has been called arachnitis, are mainly attributable to irritation of the encephalon; and here let it be well remembered, that this irritation may be induced by the diseased states of distant organs as well as by those of the investing membranes of the brain itself.

We thus see the error of designating the series of morbid phenomena, which constitute the disease of acute hydrocephalus, by the name of arachnitis.

A child suffers from indigestion and deranged stomach and bowels: severe headache, convulsions, and delirium follow. Are we to suppose that this child has an attack of arachnitis? Certainly not. It would be absurd to suppose that the stomach irradiated the diseased action on the brain through the medium of its envelopes.

The true interpretation of the case is, that an irritation of the encephalon itself has been induced by sympathy with the disturbed stomach and bowels.

Here is an illustrative case.

A child in the Rue St. Honore, four years of age, and habitually robust and plethoric, became suddenly very ill. He was constantly moaning and crying, had occasional strabismus, and at other times a spasmodically fixed state of the eyeballs, convulsive oscillation of the iris, contractions of the limbs, grinding of the teeth, and loss of consciousness. The pulse was quick and hard; and the capillaries of the face were highly injected. All these symptoms had come on very rapidly.

M. Piorry discovered that the child had been eating a large quantity of potatoes and other indigestible substances; and, on examining the stomach, he found it distended and tender; on pressing it, nausea was induced. He ordered a quantity of hot water to be given immediately, with the view of exciting vomiting and thus emptying the stomach, and several leeches to be then applied behind the ears.

The symptoms very speedily subsided, and the young patient was as quickly cured as he had been suddenly seized.

The rational interpretation of such a case is to attribute the cephalic symptoms to an irritation—arising probably from a temporary congestion—of the encephalon itself, and surely not of its investing membranes.

The most alarming symptoms, even paralysis itself, may be induced by a mere temporary fulness of the cerebral vessels, and may be as quickly dissipated by appropriate treatment.

This fact is well illustrated in the following case.

Made. la Comtesse de St. M——, residing in the Rue Vivienne, No. 8, is upwards of 70 years of age. Although tried by a succession of the most bitter misfortunes, her disposition is cheerful, her fancy lively, and her health is on the whole very good, with the exception of occasional attacks of nervous symptoms. She has been long subject to an umbilical hernia, which is but ill kept up, and during the last three years she has had several apoplectic threatenings. After dining upon rather indigestible food one day, she was siezed with a difficulty of speech, vertigo, confusion, distortion of the mouth, and powerlessness of the right arm. She soon became quite insensible, and lay in a state of apoplectic stupor, breathing stertorously, and her pulse very slow and full. On examining the abdomen, M. Piorry found the hernia large, distended, and hard. When he attempted to reduce it by firm pressure, the gesture of the patient indicated great suffering. The apoplectic state continued for half an hour; but no sooner was the hernia replaced, than suddenly the patient recovered her speech and consciousness, and soon afterwards all the unpleasant symptoms, physical as well as mental, completely vanished.

M. Piorry alludes to a similar case, which he saw along with Dr. M. Solon, and in which the apoplectic syptoms continued for a quarter of an hour, and then suddenly ceased.

He admits that in many fatal cases, where delirium, cephalalgia, and other cephalic symptoms have prevailed, we usually find traces of arachnitis, while often no lesion of the cerebral substance is discoverable. But he very justly adds, " how frequently too is the arachnoid membrane in such cases found quite healthy, and some distant viscus only exhibits signs of disease?" He therefore reverts to his former position, that there is not a single symptom, that is usually described as indicative of arachnitis, which may not be induced by disturbance of some distant organ, and more especially of some of the abdominal ones.

The cerebral irritation may prove fatal, before any appreciable lesion of structure has had time to be established; and every practitical physician knows well how often his prognosis, as to the nature and extent of the morbid change in fatal head affections has but ill accorded with the appearances found on dissection. So true is this, that the cautious practitioner will always hesitate to pronounce, with any degree of assurance, the pathological characters of any cerebral disease.

Besides this source of difficulty, it ought to be ever borne in mind that, in general, dissection reveals to us only the more confirmed and serious morbid lesions, and that it scarcely assists us in ascertaining the earlier and lest distinct changes of tissue, such as we may suppose to attend the first stages of a disease. How true is this more especially of cerebral disease! How often do we find no traces at all of morbid action, in cases too which have existed for a great length of time, and which have been accompanied with well-marked and even alarming symptoms?

In reference to the encephalic irritation of infants, Dr. Piorry is of opinion that the most frequent cause of this most insidious and dangerous malady is " une excitation pathologique" of the primæ viæ. Hence the primary importance of correcting such a disorder at its very commencement, and of counteracting its tendency to return by careful attention to diet.

Dentition also is a fruitful source of cerebral disease in infants. The proximity of the seat of pain, the increased action of the blood-vessels, and the general feverishness thus induced, will serve to explain this cause of encephalic irritation.

Internal otitis, or inflammation of the inner ear, has been repeatedly noticed as antecedent to this malady. The same may be said of the sudden retrocession of any eruptive disease on the scalp, of which the following may be taken as an example.

A girl, 13 years of age, had been long affected with tinea of the scalp. She had been once seemingly cured; but it returned with even aggravated severity. Dr. Piorry succeeded in dissipating it quickly by pursuing a rigid antiphlogistic and emollient treatment, local as well as general. A week or two after this date, she began to suffer a most acute pain over the eyebrows, and this was speedily accompanied with delirium. Fever set in; the carotid arteries beat furiously, the pulse was hard and frequent, the eyes were fixed on the ceiling, there was occasional twitching of the muscles in different parts, the face was alternately flushed and pale, and the bladder ceased to expel its urine. On withdrawing the water by means of the catheter, the young patient was much relieved; the delirium subsided, and intelligence was restored for a few minutes; but soon afterwards the stupor, alternating with convulsions, returned, the pupils became dilated, and the body perfectly motionless. She died on the ninth day after the commencement of the cephalic symptoms.

A dissection was not permitted.

In fine, as to the *causes* of encephalic irritation in infancy, we may state that the large dimensions of the head at this period of life, the tender sensibility of the whole system, the pains and distress of dentition, the development of the intelligence, the numerous new impressions made on the body, the excitable and easily-deranged state of the primæ viæ, &c.—all these influences combined will very well account for the predisposition of cerebral affections in early life.

With respect to the *symptoms*, it is unnecessary to enter upon a minute description of them. There is one, however, to which M. Piorry directs the attention of the reader with especial earnestness, as being indicative of alarming disease of the brain—he alludes to a certain involuntary rolling of the eyes, followed by a fixed turning of them upwards.

The child keeps the eyes fixed on the ceiling; he seems to be looking at something there, and yet it will be found that he sees nothing. This state lasts for a few minutes. The pupil is usually dilated for the time, and the eyelids are half open. "Before," says M. Piorry, "I commenced the use of lavements of Peruvian bark in such cases, almost all the infants who had exhibited this symptom died."

Another symptom of encephalic disease, which well deserves the careful attention of the physician, is the frequent alternation of the heat and coldness of the surface, and of the flushing and paleness of the face.

This symptom is however more frequently observed, and more distinctly marked in inflammation of the medulla spinalis, than even of the encephalon itself.

And now as to the *treatment* of this serious disease.

"In the first part of this memoir," says M. Piorry, "I have related fourteen cases of cerebral affection. In the first five, the fatal course did not seem to be at all arrested by the antiphlogistic and derivative plan of treatment which was pursued.

In the sixth one, this plan was attended with success. In the seventh, the clearing of the stomach by emetics, and then the application of leeches behind the ears proved successful.

In the four following cases, the encephalic disturbance, of long standing, and otherwise remarkable by a more or less distinct intermittance of the symptoms, was speedily removed after the employment of lavements containing Peruvian bark.

In the last three cases—and in these there had been no alternations of heat and cold—the Peruvian bark seemed to have had no effect."

M. Piorry does not however at all undervalue the importance, indeed the necessity, of antiphlogistic measures in most cases of encephalic irritation, more especially during the earlier stages of the disease. The use of leeches, cold to the head, purgatives, diuretics, &c. is quite indispensable.*

It is only when the disease has continued for some time, when the vital powers have been considerably reduced, and above all, *when there is a marked intermittence of the symptoms*—indicated by alternations of heat and chillness of the surface, of flushings and paleness of the face, and of exacerbations and abatements of the convulsions and other symptoms—that he so strongly recommends the use of lavements containing the Peruvian bark.

In alluding to the use of purgatives in encephalic irritation, M. Piorry repeats an observation, which he had previously made, that the disease is very frequently preceded by a slight degree of diarrhœa. He cautions the physician against the unguarded use of purgatives, especially of such as are acrid, under such circumstance. He assures us that he has, oftener than once, seen the cerebral affection become aggravated immediately after the action of such a medicine. The employment of emollient enemata, and a most rigid attention to the diet of the child, are all that is sufficient to correct the state of the bowels. The use of calomel, or of some other preparation of mercury, may be proper, when the intestinal evacuations are slimy, green, and offensive. On the whole however M. Piorry greatly prefers the administration of purgatives in the form of enemata.

With respect to blisters in cerebral diseases of children, M. Piorry recommends that they should not be used, until the violence of the feverish symp-

* The practice, recommended by M. Piorry, is more active and depletory than most English physicians will be inclined to adopt. One or even several venæsections, and from 20 to 50 leeches to the head are, in his opinion, the appropriate treatment of the early stages of cerebral congestion in infants.

toms has abated, and until the action of the carotid arteries and of the circulation generally is reduced. All remedies, whose action is attended with so much pain as accompanies vesication, had better be avoided in the earlier stages of the disease.

As to the application of ice, and cold lotions to the head, M. Piorry approves of them on the whole. He however very properly remarks, that, whenever the heat of the surface is below the natural standard, and the features are pale and at all shrunk, all such depressing medicaments are to be discontinued.

To revert for a moment to the use of bark injections in cerebral affections, we have already explained the circumstances in which M. Piorry recommends their use—it is only when there is a tendency to intermittence of the diseased action, or to an alternation of heats and chills, excitement and depression of the animal powers. He candidly confesses that he was indebted to M. Hippolite Cloquet for the first hint of this most useful practice.

The four cases, which he has narrated in the present memoir, are he thinks, quite decisive of the utility of the practice under appropriate circumstances.

He has witnessed its good effects in certain cerebral affections of adults. A man, 50 years of age, was subject, at a certain hour on every alternate day, to attacks of mania with a disposition to commit suicide. All the unpleasant symptoms gave way to the internal use of Peruvian bark.*

M. Piorry has not witnessed good effects from this remedy in any case, where the symptoms have been uniformly continued and have not exhibited any tendency to intermittence.

The following conclusions embody a faithful summary of the doctrines inculcated in M. Piorry's memoir.

1. During the life of a patient, it is impossible to affirm with certainty that he is affected with *arachnitis*, as all the symptoms of this disease are present in a cerebral irritation, which may be induced by sympathy with a distant organ.

2. The symptoms of arachnitis are much more frequently observed in infants and young chlldren, than in adults. We have accounted for this greater frequency, by reference to the large relative size of the head, the process of dentition, the tender and irritable state of the *primæ viæ*, &c. &c.

3. The eyes being turned upwards and fixed constantly on the cieling, is one of the most alarming symptoms of cerebral irritation in early life.

4. The primary indication in the treatment of this affection is to discover the organ, whose disturbance has given rise to it.

5. Copious depletions of blood are quite necessary in the early stage of most cases.

6. With respect to the propriety of bloodletting in cerebral irritation, we should not be guided much by the state of the pulse at the wrist, but rather by the force of the pulsations of the carotid arteries. M. Piorry dwells upon this point of practice at very considerable length. He mentions having repeatedly observed that the pulse at the wrist was small and feeble, while the carotid and temporal arteries were beating with great violence. When this state of things exists in the early stage of the disease, we need have no dread of bloodletting, either general, if the child be old enough, or local by means of leeches: in the more advanced stages, when the vital powers have been necessarily much reduced by the continuance of the morbid action as well as by the depressing remedies which have been used, we require to be very cautious in ordering further depletions.

7. After bleeding, we may have recourse to derivative applications; but we should avoid as much as possible those, which cause much pain and irritation.

8. In applying ice and cold washes to the head, attention should be paid to the removing of them, whenever the pulse becomes weak and the surface of the body chilled.

---

* Most of our readers will probably recollect the memorable case of periodic insanity in the person of Mr. M'Kerrel, narrated at great length by Dr. Johnson in one of the late numbers of this Review.

9. The symptoms of infantile encephalic irritation exhibit, in very many cases, a decided tendency to intermittence, indicated by the alternations of flushing and paleness of the face, heat and chills of the skin, &c.

10. In all such cases the use of Peruvian bark will probably be attended with benefit. The best form to administer it is in enemata. From two scruples to two drachms may be administered at a time.

M. Piorry has not found quinine nearly so efficacious as the bark in substance.

11. The best time for the administration of the bark enemata is when the face is pale, and the system is low and languid.

12. The suspected existence of gastric or enteric irritation is not to deter the physician from the exhibition of bark.—*De l'Irritation Encephalique des Enfans, by P. A. Piorry, published in the Repertoire Medico-Chirurgical, Bruxelles, Aout,* 1837.

*Remarks.* The practical physician will no doubt be much pleased with the preceding remarks on a very frequent and very dangerous malady of infants from so talented an observer as M. Piorry. We regret to find no allusion, in his memoir, to the use of opium and carbonate of ammonia in the latter stages of certain cases of cephalic irritation, or what has been too frequently called, with little or no discrimination, hydrocephalus acutus. We have often employed them with great benefit, when all other remedies, and more especially all of a depressing nature, would have been positively injurious.

It requires indeed a nice discrimination of symptoms, and not a little tact as well as decision of practice, to determine the cases, in which these powerful remedies ought to be employed. In our own experience we have been guided rather by the state of the child during its sleep, than by the presence of any particular symptoms.

When the breathing and the pulse are regular and equable, though weak and rapid, when the power of deglutition remains perfect, and when the decubitus or posture of the infant in bed is easy and natural, the cautious administration of opium, combined with an ammoniacal salt, will often save the patient. The diet at the same time must be made nutritious but light. Nothing is so good, or so easily given as beef-tea. The feet and bowels should be kept warm; the head should not be raised high by pillows, and above all the child should not upon any consideration be lifted up, as a fatal syncope is apt to supervene. If there is the slightest tendency to diarrhœa, it should be checked at once by opiate enemata.—*Rev.*

---

### M. Louis on the Value of Auscultation as a Means of Diagnosis.

(A Clinical Lecture at the Hôpital de la Pitié.)

M. Louis commences his lecture by assuring his pupils that no one can be more impressed with the high value of auscultation, as a means of diagnosis in thoracic diseases, than he is himself. He then proceeds to describe the various precautions which must be followed, if we hope to arrive at much exactitude in its employment. He shews the importance of the patient being placed in a right position, of both shoulders being level, and of the arms and neck being in an easy state of relaxation. The condition of the thoracic muscles has a marked influence on auscultatory phenomena; and the physician will be apt to commit errors, if he neglects to attend to any of these, apparently trifling, particulars. M. Louis particularly impresses upon his pupils the propriety of always ausculting the corresponding parts of the two sides of the chest, the one immediately after the other.

This rule should never be neglected. If the two sides, at any one part, emit the same or very similar sounds, there is strong reason to believe in the soundness of the lungs at these points. It is the disagreement in the sounds heard at the corresponding points of the two sides, that leads the physician to suspect the existence of mischief. We therefore repeat the advice, "*always examine the corresponding points of the thorax at the same time, the one immediately after the other.*"*

M. Louis then alludes to the position, which the physician himself should select in making his examinations—let it be easy, and without constraint or irksomeness: otherwise he can never hope to detect the less obvious sounds.

He differs from Laennec as to the preference of *mediate* over *immediate* auscultation. He in many cases prefers the naked ear to the use of the stethoscope, and assures us that he has often succeeded in discovering shades of difference in sounds, which had quite escaped his notice, when using the instrument. "Thus," says he, "the cases, in which *mediate* auscultation is to be preferred, are very rare, and all that can be found out with the stethoscope may be as well discovered without it."

*Normal Sounds.* The respiratory murmur is that light, silky, expansive sound, which is heard during healthy inspiration. It is most distinct over the anterior and lateral parts, and over the lower two thirds of the back of the chest. It approaches nearly to the blowing sound,—bruit de souffle—in the space between the vertebral column and the posterior edges of the scapulæ, on a level with the origin of the bronchi. This somewhat blowing respiration—which exists also, but in a less degree, towards the sub-spinal fossæ—is more distinct on the right than on the left side; a circumstance worthy of attention; as a person, unaware of its truth, might regard it as a sign of disease.

The larger size of the bronchi on the right than on the left side—a fact discovered by Dr. Gerhard of Philadelphia—accounts for this difference.

The respiratory murmur is heard only during inspiration. At the moment of expiration, it ceases altogether, or nearly so, except behind in the upper third of the chest. Here we may still hear a murmur, similar to, but more feeble than, that heard during inspiration.

The various sounds now described vary, as to intensity, according to the age and embonpoint of the person, and the force of his breathing; but their essential characters are much alike in all, during a state of health.

*Resonance of the Voice.* When we make a healthy person speak, while we are ausculting his chest, we hear a general resonance or echoing, which is loudest behind, between the upper and middle thirds of the chest, at the point where the respiratory murmur was perceived to be somewhat blowing. These two phenomena are owing to the same cause, and this slight bronchophony is somewhat stronger on the right than on the left side—a circumstance to be well remembered, in ausculting these parts.

*Abnormal Sounds.* The murmur of respiration undergoes various modifications in disease. The most simple of these modifications is the diminution of its strength or loudness. This occurs in pulmonary emphysema. Its degree is generally proportioned to the duration of the disease, and it is always more complete on the anterior than on the posterior part of the chest—for the emphysema is almost invariably most extensive at the former part. If it occurs over the whole thorax, and is not very considerable, there may be some uncertainty as to its real cause; for the force of the respiratory murmur varies a great deal in different persons. But if it is limited to one side, or to one part of the one side, and is found to exist, more or less distinctly, on several repeated examinations, we have reason to suspect that it is attributable to some pathological cause. Besides pulmonary emphysema, pleurisy, phthisis and pulmonary catarrh often induce a very decided diminution of the respiratory murmur. In these diseases however, the diminution of its strength is never the only symptom; it is always associated with other more characteristic changes.

---

* The same good advice is much insisted upon by Dr. Stokes in his recent work on the Diseases of the Chest.

In pleurisy, when the effusion into the thorax is not very great, we may still perceive the murmur through the effused fluid; but it is always much less distinct than over the corresponding part of the opposite side. The weakened murmur is perceived to be, as it were, deep-seated. It is usually feeblest over the inferior and posterior parts of the chest; whereas in emphysema, as already noticed, it is so anteriorly and superiorly. Besides, in this disease the murmur is heard to be more superficial, and has a dry sound, having lost " sa souplesse ordinaire."

In phthisis pulmonalis, it is over the upper and fore parts of the chest that the diminution is always most decided.

In catarrh, it is limited and not permanent; and, as it depends, in this disease, on the obstruction of the small bronchi with mucus, the murmur is always most distinct after a sharp fit of coughing.

Thus the mere diminution of the intensity of the respiratory murmur may, independently of the presence of any râles, lead to the diagnosis of several affections of the lungs; not with much certainty indeed, but at least in such a manner as to afford very strong presumption of their existence. In studying this symptom, we should always pay much attention to its seat or localization, its persistance, its degree of proximity to, or distance from, the ear of the auscultator, and to its dryness or its pliancy.

*Absence of the Respiratory Murmur.*—Sometimes the murmur is quite inappreciable over a greater or less extent of one, or partially of both sides of the thorax. Such a state may exist when a large tumor presses on the more considerable bronchi, as in some cases of thoracic aneurysm, or when there is an extensive effusion of air or water into the cavity of the chest. In these two last cases, auscultation, independently of other means of exploring the chest, is attended with considerable uncertainty: percussion is then of the greatest importance.

*Alteration of the Respiratory Murmur.*—One of the most remarkable changes is the "respiration amphorique," or " bourdonnement amphorique," heard when the air enters a large cavity through a narrow orifice. Its presence leads the physician to suspect either a large cavity in the pulmonary substance, caused by the melting down of tuberculous matter—and in such a case the sound is always to be heard towards the summit of the lungs—or by gangrene of some portion of the lungs, or an enormous dilatation of one or more of the bronchial tubes. In estimating the importance of this auscultatory sign, much will depend on the situation where it is heard. If heard towards the apex of either lung, there is at once reason to suspect a tuberculous cavity. Still, however, it is possible that one of the large bronchi may have become much dilated at this point, and that the pulmonary tissue itself is quite sound. The adroit use of percussion, along with due attention to what are called the *rational* symptoms of the case, will speedily remove all uncertainty.

*Bronchial Respiration* is one degree less than the "amphoric blowing." It is quite analogous to, or rather identical with, the sound which we hear if the ear is applied over the trachea of a healthy person. Bronchial respiration exists whenever the air passes through the bronchial tubes, without reaching the pulmonary cells.

In the cases where the aërial tissue of the lungs changes its condition, and becomes more or less solidified, bronchial respiration is one of the most certain signs of the red and grey hepatisation of the pulmonary structure—those lesions which constitute the second and third stages of pneumonia.

They are generally found posteriorly, and much more frequently near the base than towards the summit of the lungs.

2. This auscultatory sound is present also in some cases of pleurisy; but then only in an indistinct and masked degree: besides, it is audible only at one part of the chest; this part varying with the quantity of the effused fluid, and the position of the patient.

3. Dilatation of the bronchial tubes is another disease, in which this sound is heard. Then, it is always persistent in the same place.

4. Bronchial respiration is one, among other signs, of the presence of unsoftened tubercles. It is, therefore, always a suspicious symptom when we have reason to suspect a tendency to phthisis.

The unpractised auscultator must be on his guard, however, not to confound the ordinary respiratory murmur, when rather louder and rougher than usual, with the true bronchial respiration. Experience alone will enable him to avoid this mistake. In this, as indeed in all cases, much assistance will be obtained by examining most attentively first the one side, where the sound is heard, and then immediately afterwards the corresponding part of the other side; remembering, all the while, that in the healthy state the respiratory murmur is usually rather louder and somewhat more dry and bronchial on the right than on the left side.

In all the cases, now mentioned, in which the bronchial respiration is heard, the organic alteration of the lungs presents a certain similitude. The pulmonary tissue is condensed by inflammation in pneumonia, and by the compression of the effused fluid in pleuritis; while, around dilated bronchi, it is always more or less indurated; and in phthisis the air-cells are occupied with the hardened tuberculous matter.

The phenomena of expiration are usually more or less affected in all cases, where the bronchial murmur is heard. It is more prolonged and blowing than in health. To judge aright of the value of this sign, we must remember that expiration, which in most persons takes place without any audible murmur, may nevertheless be accompanied by a slight bruit, without being indicative of any disease — provided always the sound be nearly equally distinct on both sides.

At the same time that the expiration becomes prolonged, the inspiration loses its soft and silky murmur, and assumes a rougher and coarser character. These two phenomena add mutual importance, the one to the other. When, therefore, both are perceivable under the clavicles in a delicate young subject, we have strong presumptive grounds to fear the existence of tuberculous disease. Let it, however, be well remembered that our apprehensions may be thus awakened, ere yet the disease has made great progress, and while it is still a good deal under the control of the healing art.

Between bronchial respiration and the amphoric blowing sound, auscultators admit another, to which the name of *cavernous respiration* has been given. Whenever this sound is heard, we may be quite assured that there is a cavernous excavation in the pulmonary tissue communicating with several of the bronchi.

Now such an excavation may be produced in four different manners.

1. By the melting down of tuberculous deposits. When this is the case, we always find the cavernous respiration towards the summit of the lungs. The place or situation, therefore, indicates the nature of the existing lesion.

2. In the progress of a pneumonia, when an abscess or vomica is formed—a termination, it must be acknowledged, of very rare occurrence—the cavernous murmur becomes audible at the time that the abscess first communicates with any of the open bronchi.

In such cases, as those now alluded to, this auscultatory phenomenon is usually heard towards the base of the lungs.

3. An excavation in the pulmonary substance may be produced by partial gangrene — the gangrenous portion being melted down, and then either absorbed or discharged into the adjacent bronchia.

4. It may be the effect of an enormous dilatation of some of the bronchi.

In the two last-mentioned cases, the mere *locality* of the cavernous respiration does not, as in the two former, assist the physician in his diagnosis of the existing lesion. In phthisis, it is almost invariably perceptible towards the apex of one or of both lungs; and in vomica from pneumonia it is most frequently detected near their basis. On the other hand, when it is the result of gangrene or of dilated bronchi, there is no part where it is oftener heard than another. We must, therefore, trust to some of the other symptoms to assist us in our diagnosis.

*Modifications of the Resonance of the Voice.*—The most remarkable of these modifications is that one which is known by the name of *bronchophony.* It always accompanies bronchial respiration. Whenever, therefore, this is heard any where, we have only to cause the patient to speak, while our ear is applied to his chest, and the sound called bronchophony will be heard.

The one necessarily goes along with the other. They are discoverable in—

1. The first and second stages of pneumonia.

In this case the bronchophony is constant, remains for several days, and varies in extent and intensity, according to the severity of the existing disease.

2. Dilatation of the bronchi.

In this affection it has not always the same distinctness and force—depending on the greater or less degree of the induration of the pulmonary texture, around the dilated bronchial tubes.

3. In tuberculous deposits.

When it proceeds from this cause, it is always heard towards the summit of the lungs, under the clavicles, or in the supra and sub-spinal fossæ of the scapulæ. The remarks which we have made on this subject, when treating of bronchial respiration, are quite applicable here.

Thus the farther we advance in our examination of auscultatory phenomena, the more we become satisfied of the great importance of attending to the *situation* where they happen to be detected—the same sign belonging to several lesions, which differ not only in their essential character, but also in their locality.

4. In pleurisy the bronchophony manifests itself at the posterior and inferior part of the chest; which circumstance alone would not be sufficient to distinguish the nature of the affection, but which, when taken in conjunction with another character, will go far in determining this point. This circumstance is the possibility of its displacement or change of locality with the effused fluid, at least during a certain length of time. This displacement, however, is not absolutely necessary to render the diagnosis certain, when the bronchophonic sound assumes that peculiar modification, known by the name of *œgophony.*

We may admit, as a general rule, that œgophony, variable though it be in intensity and often not of easy detection, is truly pathognomonic of pleuritic effusion—provided this effusion be in moderate quantity, neither very scanty nor very abundant.

But let it be remembered that neither the locality of the bronchophony at the lower and back part of the chest, nor the possibility of its displacement by change of posture, is to be regarded as an absolute and invariable sign of pleurisy. To satisfy you of this truth, I need only mention that Laennec has seen more than one case of numerous detached adhesions between the opposite pleuræ; these adhesions isolating one effusion from another. In such circumstances it is evident that an effusion may be found suspended, so to speak, at different elevations, each effusion too being quite incapable of any displacement.

Another difficulty sometimes presents itself. In certain subjects the pleuritic pain is very inconsiderable, the resonance of the one side of the chest, on percussion, is not very different from that of the other, the œgophony is indistinct, and the only auscultatory phenomenon, that is very manifest, is a slight abatement of the respiratory murmur. In such a case, are we to suppose that there is any effusion? If there is indeed any, it must be in a very trifling quantity.

With respect to the etiology or rationale of œgophony we must acknowledge that it is not very obvious.

Laennec attributed it, in a great measure, to a certain degree of *flattening,*

which the bronchi experienced from the compression exercised upon them by the effused fluid. In addition, however, to many other arguments against this doctrine mentioned by Laennce himself, there is another which may deserve notice. When strong and thick adhesions have taken the place of the effused fluid, the flattening of the bronchial tubes may still be supposed to continue, and the œgophony therefore should be heard as before; which is not the case. We are not prepared with any interpretation of our own to propose; and we shall, therefore, quit the subject, with merely repeating that œgophony has a marked resemblance to bronchophony, differing from it only by a peculiar modification; viz. the bleating sound.

Another variety of bronchophony is the sound, designated by the name of *pectoriloquy*—a phenomenon similar to that, which would be perceived if the patient spoke directly into the ear of the auscultator. The indispensable condition of the occurrence of this very peculiar sound is the existence of a cavity in the pulmonary tissue, communicating with one or more of the bronchial tubes. It is met with in phthisis, when the vomicæ have acquired a certain dimension, and are surrounded with an indurated tissue; in abscess of the lung succeeding to pneumonia; in gangrene of the lung; and in enormous dilatation of a bronchial tube.

We need not again point the reader's attention to the importance of the seat or locality of the pectoriloquy, as in some degree indicative of the lesion which has produced it. It not unfrequently happens that even a large cavern may exist in the lungs, and yet no pectoriloquy is ever audible. Such is the case when there is no communication between the cavern and any considerable bronchus, or when it is in a great measure closed up.

*Of the Râles.* The auscultatory signs, which we have hitherto described, are, all of them, merely morbid or abnormal modifications of the respiratory murmur. We shall now examine another series of phenomena, which are no less important, but whose "point de depart" is not to be founded in the normal state of respiration. This series comprehends the *râles*, which have been distinguished into two classes, the dry and the moist.

The former—the dry *râles*—may be reduced to the two principal ones—the sibilant, and the sonorous *râle*.

The sibilant *râle*, may be compared to a fine prolonged whistle, grave or acute, dull or clear. It is found to occur in—1. Emphysema over a greater or less extent of the thoracic parietes. When strong, it sometimes quite conceals the respiratory murmur. 2. Catarrh is another disease in which it occurs—then it is usually more limited, and is often observed to move from one point to another; a circumstance which is not apt to take place in pulmonary emphysema. 3. The sibilant râle is also met with in a large proportion of typhoid cases. M. Louis says, "it occurs in three-fifths of the cases, usually about the eighth day of the disease, and over the whole extent of the chest"—[dans les affections typhoides, il se manifeste dans les trois cinquiemes des cas ordinairement vers le huitieme jour, et dans toute l'etendue de la poitrine.] Some physicians have regarded it, under these circumstances, as indicative of inflammation of the bronchi. The quickness, however, with which it frequently ceases, is opposed to this conjecture; and it is altogether more rational to attribute it to the presence of a small quantity of fluid in the bronchial tube.

The *sonorous râle* is a grave and often noisy blustering sound, resembling sometimes a loud snoring, at other times the cooing of a dove, or the booming of a bass violin. It usually is found at the commencement and during the progress of a pulmonary catarrh. Laennec attributed it to the contraction of the bronchi, especially at the angles of junction of any two of them, in consequence of the tumefaction of their lining mucous membrane.

The *moist râles* are of more frequent occurrence than those of a dry character. They are also, on the whole, more important as signs for diagnosis, and may often, *per se,* suffice to distinguish many pulmonary affections. One of the most common is the—

*Mucous Râle.* It has been compared to the noise, which is made when air is blown through soap-suds, or any viscid fluid. This râle is heard in—

1. Pulmonary catarrh : it then exists on both sides of the chest, and progressively descends.

2. Phthisis, when the tubercles have softened, and their contents begin to be evacuated into the bronchial tubes.

3. Pneumonic abscess, pulmonary gangrene, and dilatation of the bronchi.

The mucous râle is usually confined to one side: by itself, it cannot be regarded as a pathognomonic sign of any disease. There are several shades or varieties of the mucous râle, according to the size of the bullæ caused by the air passing through the mucous fluid.

*Crepitant Râle* has been aptly compared to the crackling noise, which is heard when we throw salt on a live coal, or when we rub a piece of dry parchment between the fingers. It exists in one disease only, pneumonia, and is truly pathognomonic of it, or rather of its first stage—that of pulmonary congestion or *engouement.* It is a fine clear sound; and when it exists, we rarely can hear any distinct respiratory murmur.

Although the crepitant râle is pathognomonic of the presence of pneumonia, yet this disease may exist, and no such râle be heard. This therefore is a most important fact, and should never be lost sight of by the practical auscultator. When the rational symptoms of pulmonary inflammation exist, the absence of the crepitant râle is not to sway our diagnosis to the contrary.

MM. Andral and Chomel have particularly insisted upon this point. In numerous cases have they found that, when the pneumonic inflammation was deep-seated in the substance of a lung, and confined to a few of its lobules, the crepitant râle is often not at all discoverable.

There is another circumstance respecting this *râle,* which deserves to be well known to the physician. In some persons, in a state of perfect health, it is audible over every part of the chest, when they take a deep inspiration for the first time—after which, it altogether ceases. Is this owing to the presence of a small quantity of mucus in the small bronchi, or to the extension (deplissement) of their lining membrane? This latter conjecture will perhaps be deemed the more probable, if we consider that the sound is quite analogous to that heard in a new-born infant, when the air enters for the first time into the chest.

There is a variety or modification of the crepitant râle, distinguished by the appellation of the *sub-crepitant râle.*

The former is a finer, clearer, drier, and a more equal and uniform sound ; the latter is coarser, and more humid, approaching to the character of the mucous râle. This distinction is of great importance. The one is pathognomonic of genuine pneumonia, the other of pulmonary catarrh.

The subcrepitant râle is heard especially in—1. Acute pulmonary catarrh: its constant seat in this disease is the posterior and inferior part of the chest, usually on both sides at the same time ; sometimes it extends upwards ; but at first it is always heard lower down towards the base of the lungs. It varies according to the stage or progress of the catarrh, and rarely masks completely the respiratory murmur.

Laennec has stated that the subcrepitant râle is one of the chief signs of emphysema. There is some doubt as to the correctness of this statement, for this reason ; the seat of pulmonary emphysema is almost always in the upper and anterior part of the lungs, and there, therefore, the râle should be heard ; whereas such is seldom or never the case.

2. Pulmonary œdema, according to Laennec. M. Louis does not seem to assent to this assertion.

3. If the subcrepitant râle is heard on one side only of the chest, at the lower and posterior part, it is indicative of either tubercles or more rarely dilatation of some of the bronchi. There is no exception to the accurary of this remark. Of between five and six hundred cases of pulmonary catarrh, which I have examined, says M. Louis, during the last five years, the subcrepitant râle was heard on both sides of the chest in every one of them without exception.

4. It is heard not unfrequently at the upper part of the chest, and then, of itself, it is almost a sufficient index of the existence of tubercles.

You have lately seen, Gentlemen, a case, very illustrative of this point, in a woman affected with chronic peritonitis: in her, there was a subcrepitant râle audible at the upper part of the chest, and there only. This symptom confirmed the opinion which I had already formed, that the disease was of a tuberculous character.

I may mention, en passant, that I have never met with a case of chronic peritonitis, which has not been more or less obviously of a tuberculous origin.

Having thus shortly discussed the various râles, we shall now allude to another auscultatory sound, known by the name of *metallic tinkling*. It is a clear, silver sound, exactly similar to that produced by the dropping of a pin or small-pointed object on a glass or metal vessel.

It is heard in two cases only, viz. when a large excavation exists in the substance of the lungs, or when there is a perforation at some part of the pulmonary pleura, so that there is a communication between the air-tubes and the cavity of the pleura.

For the production of this sound it is necessary that there be a pretty considerable cavity, containing a small quantity of fluid and a large quantity of air. Laennec accounted for this very peculiar sound by attributing it to the falling of a drop of fluid, usually purulent, from the upper to the lower part of the cavity. Of late years M. Beau has suggested another interpretation. He is of opinion that it is produced by a large bubble of air rising through the contained fluid and bursting on its surface. In support of this explanation, he states that he has never met with the metallic tinkling in any case of perforated pulmonic pleura, where the orifice was above the surface of the fluid in the pleural cavity.

The last auscultatory sign, which we shall notice, is that which has been called the *rubbing sound* (bruit de frottement). It is a valuable diagnostic sign; but often very difficult of detection, and one which the inexperienced auscultator may very possibly be deceived in, if any clothing intervenes between his ear and the patient's chest.

It is heard occasionally in—1st. Interlobular emphysema of the lungs.

2. In the very early stage of pleurisy, before the process of effusion has commenced, and also towards the very close of the disease, when the effused fluid has been re-absorbed, and nothing remains but false membranes.

3. Lastly, in some pneumonic cases which are almost entirely cured, there is heard for a long time a very distinct crepitating sound; which, coinciding with a certain degree of obscurity in the respiratory murmur, may impose on the auscultator, and make him believe that there still remains an *engouement* or inflammatory congestion which cannot possibly exist.

It is probable that the sound is owing to the rubbing of the false membranes, as yet very imperfectly organised. The reticulated texture of these membranes, when one is rubbed against another, may very probably give rise to the persistant crepitating sound.

Such seems to me the interpretation of this auscultatory phenomenon.—*La Presse Medicale.*

---

### HÔPITAL NECKER.

(Clinical Lectures of M. Bricheteau.)

### PHTHISIS PULMONALIS.

*Pathology of Tubercles—Treatment by Emetics and Issues.*

During the present year, 1837, the number of phthisical patients in Bricheteau's wards has been, as usual, very great.

This physician has, for a series of years, paid minute attention to the pathology of this most destructive disease, and more especially to the anatomy of tuberculous deposits in their primary or earliest stages. He acknowledges his obligations to M. Rochoux, who seems to have set the example of studying the phenomena of these formations from the period, when they are first appreciable by the senses.

He (M. Rochoux) is of opinion that tubercles at first consist of corpuscles or granules, which are similar to those we observe in the buffy coat of inflamed blood. These corpuscles he has described to be of about one-tenth of a line in diameter, of a nacreous colour, and susceptible of assuming all the shades of hue intermediate between grey and rose-colour.

They are of a softish consistence, but are not diffluent. If we cut a mass of such tubercles across with the scalpel, the surface of the section is found to be homogeneous, and free from the admixture of any other tissue. At a later stage of the formation, they become rounded and circumscribed; they are attached to the pulmonary tissue by a number of loose filaments, which form an envelope, like a spider's web, around them.

When extracted from the pulmonic tissue with the point of a sharp instrument, a tubercle, in its earliest stage, surrounded with its filamentous covering, seems to lose itself in the midst of a cloudy opacity, when it is immersed in water. It again becomes visible when taken out, the shaggy filaments falling together as before.

When the tubercle has attained the diameter of the tenth of a line, its colour usually changes somewhat. Its shining gelatinous aspect gradually changes into a greyish, or greyish-yellow hue.

The phenomena, which we have now described, are visible to the naked eye; but they are rendered much more distinct with the use of a small microscope.

If confirmed by future observers, these observations clearly shew that the opinion of Magendie, Cruveilhier, Andral, and Carswell—all of whom maintain that tuberculous matter is at first in a fluid state—is incorrect.

The doctrine, that there exists at first a minute deposit of serum or of blood according to some, and of hydatidic vesicles according to others, is equally fallacious.

If asked, what is the real nature or composition of a tubercle at its very earliest commencement, we must answer, says M. Bricheteau, with M. Rochoux, that there exists an atom of vitiated fluid (un atome de liquide vicié)* which combines with the already-diseased tissue of the lungs, and ultimately changes their texture. It may be, if we choose so to say, an alteration of nutrition, of *hematosis*, which ultimately produces a degeneration of tissue.

In all probability, the morbid change commences in the inter-lobular cellular substance of the lungs.

M. Bricheteau confesses that he is not acquainted with any symptoms, indicative during life of this, the earliest stage, of tuberculous formation. He is still busily occupied with researches in this most important subject, and proposes ere long to publish the results in a separate work. The remainder of M. Bricheteau's memoir is occupied with an account of the *antimonial treatment*, as he terms it, which he has found to be very useful in the earlier stages of phthisis.

During the whole course of the present year, 1837, except in the warmest months July and August, several phthisical patients were daily submitted to the treatment by minute doses of emetic tartar. In general, young subjects, in whom the disease was in its first or second stage, were selected for the trial. A few however in the more advanced stages were also subjected to this medication.

In every case, without exception, the patient confessed himself relieved at first by this mode of treatment. Some became soon tired of it and refused to persevere; in others diarrhœa super-

* There appears to be some incongruity between this statement, and one, made a few lines previously, that tuberculous matter is not at first in a fluid state.—*Rev.*

vened; but the greater number were able to continue its use for a considerable length of time.

In many, the sweats and expectoration were very promptly mitigated; or the pains in the chest were relieved, and all the symptoms were much ameliorated. In a few, the medicine did not appear to exert any decided effect. We may safely assert that the greater number received very decided benefit from the antimonial treatment, even when the disease had made great progress. The following may be adduced as an example in point.

A young man, 17 years of age, had been sent to Paris in a forlorn state. The existence of a cavity in the right lung was acknowledged by all, who examined him. He remained in the hospital for nearly a twelvemonth; during which period, at different intervals, he took the antimonial mixture, and had several issues (made by the actual cautery) established under the right clavicle and below the scapula on this side. He returned home perfectly well, and continued so for four months—the date of his being last seen.

Another patient was a washerwoman, 38 years of age. The disease was still in the first stage, when she was subjected to the treatment by the emetic in small doses (l'emetique à petites doses), and by deep cautery issues established below the clavicle, on the diseased side.

A third successful case occurred in a semstress, 30 years old, and mother of several children. The signs of confirmed tuberculous disease of the right lung were unequivocal; cough, purulent expectoration, the gurgling sound, (gargouillement), and night sweats. In less than a month, under the antimonial medication, her health was greatly improved; the sweats had ceased; the cough and expectoration were nearly gone, and the patient had recovered her strength and embonpoint. When she left the hospital, she was "dans un etat très satisfaisant;" but M. Bricheteau was anxious to have induced her to stay longer.

The last case was still more satisfactory, as to the results of the antimonial treatment.

The patient was a female, 60 years of age. There was a distinct pectoriloquy heard over the upper part of the right side of the chest: in addition to this auscultatory sign, all the usual rational symptoms of phthisis were present. The antimonial mixture administered to this woman, for 15 days consecutively, never failed to produce, at each dose, two or three fits of vomiting. The effects of this medication were so decidedly beneficial, that the most distressing symptoms of the disease were speedily much mitigated; the cough, expectoration, and general feebleness became greatly less; the appetite returned, and the gurgling, heard over the summit of the right lung, ceased to be audible. When she left the hospital, six or seven weeks after the date of her admission, her condition was on the whole highly satisfactory.

The antimonial mixture, used by M. Bricheteau at the Hôpital Necker, contains from one to three grains in five ounces of water and one of syrup. A large tablespoonful of this mixture is usually given morning and evening; but, if this quantity produces no effect, another spoonful is administered in a quarter of an hour after the first dose. If diarrhœa is brought on, pills composed of ipecacuan and digitalis powders are used. The diet consists of rice and other "potages" made with milk; the ordinary drink is milk, diluted with any farinaceous decoction.

In lecturing upon the employment of the antimonial medication in pulmonary phthisis, M. Bricheteau mentions that many years ago he attended, along with MM. Portal and Hallé, a young lady who had been sent to Paris for the benefit of metropolitan advice. The impression on the minds of all was, that she could not possibly recover; so far advanced was the disease. The doctors, we are told, were politely dismissed, and a quack was called in. He gave the patient, for several days successively, an emetic. It was astonishing what benefit this treatment seemed to produce at first: all the symptoms were much mitigated for a short time; although the patient died quite as soon as if no means had been used, the disease being in its third or last stage.

M. Bricheteau, however, drew a useful hint from the circumstances of this case; and ever afterwards he has been in the habit of resorting to the treatment, described in the preceding page. —*Gazette des Hôpitaux.*

*Remarks.*—It is really very annoying to find the descriptions, given by French writers, of diseases and therapeutic means so obscure and unsatisfactory as they usually are.

Much of the preceding memoir is, to say the truth, far from being intelligible. It is rather from accidental intimations or allusions, than from positive statements in it, that we are led to infer what the treatment, pursued by M. Bricheteau consists in. We were uncertain at first whether the antimonial was given as an emetic, or merely as an expectorant and diaphoretic, until we came to nearly the end of his paper. We now understand that he employs it to excite vomiting.

The treatment of the earlier stages of pulmonary consumption by the frequently-repeated use of emetics is certainly a very rational and often too a successful plan. In this country, however, the sulphate of zinc or of copper is justly preferred to the use of any antimonial preparation. The former medicines are much less apt to impair the energies of the system, or to disorder the intestines, than the latter.

Along with the use of emetics, the establishment of a caustic issue over the suspected seat of the disease, the breathing of a pure mild air, and the use of a nourishing but not stimulating diet, will be found in most cases of decided benefit. No faith has ever been, or is likely to be, attached in this country to the foolish and utterly unprofessional doctrines of such a writer as Dr. Ramadge; although we observe by the French journals that he has imposed, for a time, on the credulity of our continental neighbours by his bold assertions. Even they are now beginning to find out their mistake, in listening to such an adviser.—*Rev.*

---

### Influence of Climate on Phthisis Pulmonalis.

Every one is aware that it has been the practice for many ages to send consumptive patients to warm climates. The recent researches of M. Costallat have very satisfactorily shewn that the genuine tuberculous disease of the lungs is of rare occurrence in tropical countries. We believe that the truth of this remark is confirmed by most practitioners in southern latitudes.

In the recent edition of M. Laennec's immortal work, edited a year ago by M. Andral, there is a very useful statistical summary of the best-known facts on this important subject.

In one of the most northern countries of Europe, Sweden, and more particularly in its metropolis, the mortality from pulmonary phthisis appears to be very small—not exceeding 63 in every 1,000 deaths. In London, on the other hand, it is calculated that 236 of every 1,000 deaths is caused by this destructive disease. What an appalling mortality!

Dr. Chrichton found that phthisis is infinitely more frequent in Great Britain, than in the northern parts of Russia.

In the temperate regions of Europe, comprised between the fifty-fifth and forty-fifth degree of latitude, phthisis is much more frequent than it is further to the northward.

Thus throughout Germany, and more especially at Berlin, Munich, and Vienna, it is much more destructive than at St. Petersburg and Stockholm. In Paris and London it is still more common and fatal. While at Vienna and Munich its ravages amount to an eleventh or tenth of the whole mortality, and at Berlin only to a fifteenth, they equal a fifth in London, and nearly the same proportion in Paris.

In the southern parts of Europe, from the forty-fifth to the thirty-fifth degrees of latitude, pulmonary consumption is still found to be a very common disease; and, in this region, there are said to be some places, where it is even more frequent than in the north. Thus it has been calculated that at Marseilles it causes nearly one fourth of the entire mortality; at Geneva, a sixth; and at Naples, an eighth.* On the other hand at Rome, the mortality has been estimated not to exceed one-twentieth of the whole.

At Madrid, Lisbon, Gibraltar and Malta the deaths from phthisis are very numerous. Even between the twentieth and tenth degrees of northern latitude, it does not cease to shew itself. Thus it is tolerably frequent in the Antilles.

From these statements it appears, that pulmonary phthisis is a disease which is found more or less frequent in almost all climates; that a cold temperature is not essentially and *per se* favourable to its development; and that in some warm climates it is more frequent than in those farther to the north.

M. Berge (the author of the article from which we have drawn these remarks) comes to the conclusion that a residence in a warm climate, *where the temperature is steady and not subject to great variations,* is certainly beneficial to those persons who have a phthisical predisposition, or in whom even the early stage of the disease has manifested itself; but that it is rather injurious than otherwise to those, in whom the morbid changes of the lungs are far and seriously advanced.

The statistical researches of Drs. Renton and Heniken of Madeira may be adduced in support of the accuracy of his statement.

These two talented physicians unhesitatingly declare, that a residence in that island will often prevent the development of the disease before it has fairly shewn itself, but that it cannot cure the evil, when once established.

The following table, drawn up by the former of these physicians, will be found instructive.

Of 47 patients in confirmed phthisis—

32 died within six months after their arrival on the island.

6 died on their return to Europe.

6 remained on the island, and died.

3 fate unknown.

—

Total 47

Of 35 cases of incipient phthisis—

26 were apparently cured, when the patients left the island. They remained quite well, when last heard of.

5 were benefited; but were subsequently lost sight of.

4 died.

—

Total 35

It is now an acknowledged truth that the change to a warm climate, when the disease is far advanced, is most injurious. It generally accelerates the fatal termination.

Almost every British practitioner, who has resided in a tropical country, admits that a hot climate aggravates all the symptoms, after a certain period of the malady.

Dr. Ferguson of Jamaica very pointedly alludes to this, in describing the diseases of that island.—*Journal des Connoissances Medico-Chirurgicales.*

*Remarks.* We are induced to append a few remarks to the preceding, as the subject is one of great practical interest to the physician. We shall first allude to the climate of the South of France; as, for a great length of time, this district held a high, but, as we shall find, a most unmerited repute for the salubrity of its climate to phthisical invalids.

The entire district from Montpelier eastward to the boundary of Piedmont is subject to great vicissitudes of weather. The heat is often intense, and irritating from its dryness, during a great length of time. A bitter cold North-East wind (mistral) may then quite suddenly set in, and continue to blow for several days successively; so that a fire is even necessary in the middle of Summer. Such a climate, it is obvious,

* Perhaps the writer includes in this estimate all the patients sent from England and other countries, who may chance to die at these places.

must be most hurtful in all pulmonic diseases.

It was altogether a most singular error—an error which remained so long undisputed—that physicians should ever have selected Montpelier, as a residence for phthisical patients.

It has completely lost the repute which it enjoyed for so many years; more especially from its having been, of late years, well ascertained that phthisis is by no means an unfrequent disease even among the natives of the place.

The climate of Marseilles, although somewhat more favorable than that of Montpelier, is liable to the same objections. It cannot therefore be a suitable residence for consumptive patients.

That of Hyeres is decidedly much better. On the whole, perhaps this is the best place in the South-East of France which can be selected.

With regard to Nice, Sir J. Clark and other observers agree that, although its climate is more genial than that of most places on the South Coast of France, in consequence of its being protected from the cold northerly winds by the high range of mountains behind, it is far from being a desirable residence for invalids suffering from pulmonary complaints. The air during the warm months is exceedingly dry and irritating; and hence, whenever there is a disposition to inflammatory action, it cannot but be unfavourable.

With respect to the climate of Italy, in reference to its influence on phthisical disease, we find that, until of late years, very erroneous ideas have prevailed among medical men. It used to be the custom to send patients, threatened with or actually suffering from tubercular affections of the lungs, to Italy; without distinguishing those places which are proper, from those which are now known to be positively injurious.

Within the last twenty years, a great change has been effected in this respect; and we do not now hear of such unwise and indiscriminate advice being given by any medical man. The climate of Italy is much better suited for many other diseases than for pulmonary consumption; except perhaps for the very earliest stage of this malady. As Summer residences, the baths of Lucca, Sienna, or the neighbourhood of Naples, are perhaps the best which can be chosen.

As Winter abodes, Rome, Pisa and Naples are usually preferred. The climate of Rome is less dry and irritating than that of Nice or of Provence. It is said that about one third more rain falls at Rome than in the latter country, and the number of rainy days is considerably greater.

Sir J. Clark says that he is inclined to prefer the climate of Rome to that either of Naples or of Pisa, when the patient can take exercise in the open air. On the other hand, when it is necessary for him to be kept from much exposure, perhaps no place in Italy is equal to the neighbourhood of Pisa.

The climate of Naples is more subject to winds, and its atmosphere is rather more exciting, than that either of Rome or of Pisa. It is, on the whole, not so well suited for phthisical patients as they are.

One great evil of the climate of Italy, in almost every part of the Peninsula, during the Summer months is the sirocco wind, so enervating and exhausting to all whose health is delicate.

None of the islands in the Mediterranean presents a very desirable residence for phthisical invalids. The want of suitable conveniences and comforts in most of them is a great objection; and in Malta, which is perhaps the only one to which this does not apply, the climate is far from being favorable According to Dr. Hennen, the mortality among the natives from consumption is very great; amounting, it is said, to nearly one tenth of the whole.

We shall now briefly allude to the climates of the islands in the Atlantic, which have been recommended for consumptive diseases.

In almost every respect, Madeira is to be preferred. Although its mean annual temperature is only about six degrees higher than that of the South of France and Italy, this temperature is much more equable and uniform through the different seasons of the year. Thus, while the Winter is twelve degrees warmer, the Summer is nearly five degrees cooler—the range of temperature being thus little more than one half of what it is in the most favoured spots of the South of Europe.

In steadiness too of temperature from day to day, Madeira is a most favoured climate. It is found, moreover, that it is nearly as humid as the climate of Rome, although the number of rainy days is much fewer—being in the one place 73, and in the other 117.

It is almost unnecessary to allude to the climates of the Canary or of the Azores islands; as they have no advantages over that of Madeira, and, in other respects, the conveniences of residence are greatly inferior.

We shall now briefly allude to those parts of our own island, of which the climate is considered to be most favorable to phthisical patients. They are all situated on the South or South-West coast. In Cornwall, the neighbourhood of Penzance and of Flushing; in Devonshire, Torquay, Dawlish, Sidmouth, and Salcombe; Hastings in Sussex; and Undercliff in the Isle of Wight, have all acquired a reputation for the comparative mildness and salubrity of their climate. Perhaps the best are Penzance, Torquay, and Undercliff. The climate of the first of these places is remarkably equal and uniform throughout the year; the annual mean variation not exceeding 18°, whereas in London it is at least 26°. The chief objection to Penzance is the extreme humidity of the atmosphere, and the frequent recurrence of very high boisterous gales. The quantity of rain which, it is said, falls at Penzance is nearly double that which falls in the metropolis; and the number of rainy days is much greater. From these causes, and from its exposure to the north and north-east winds, the climate is colder during the Spring, than either at Undercliff or at Torquay.

One very great advantage of Torquay is the considerable extent of sheltered country in the neighbourhood; thus the patient is enabled to take exercise in the open air, from whatever direction the wind is blowing. Its position also, observes Dr. Clark, on the southern declivity of a range of pretty steep hills, composed chiefly of calcareous rocks, renders it comparatively dry.

Before quitting the subject of British climate, we may allude to the Channel Islands, as they are occasionally resorted to by invalids.

On the whole this climate resembles that of Devonshire, although it is somewhat warmer, and more exposed also to high winds. During the Spring months there is often a long continuance of cold north-easterly winds, which are perhaps the most trying of all to patients suffering from pulmonic complaints.

The Summer season is generally very delightful. Jersey is in all respects the island, which should be preferred.

---

CASE OF ACUTE PHTHISIS—ANALYSIS OF THE CASES REPORTED IN M. LOUIS' WORK — REFLECTIONS ON THE SUBJECT, by Dr. D'ESPINE, of Geneva.

Josephine Polian, 20 years of age, was seized in 1836 with an acute disease, which obliged her to take to bed on the 31st of December, and proved fatal on the 26th of the following month, January.

The reporter goes on to give the reader a history of his patient's father and mother, the number of family they had, how long she (the patient) was nursed, what diseases she had in infancy, where she was educated at first, how long she remained at the different schools, how she had a nasal catarrh for several months when she was seven years old, how porrigo of the scalp supervened, when the catamenia appeared, and lastly, that she never had suffered from hæmoptysis, rheumatism, nor leucorrhœa!

This is all quite after the newest German fashion; but, as the information is not likely to amuse the English reader, we shall pass it by.

All that appears to us necessary to be mentioned is simply, that the girl was of a tender constitution during her early years, and had rather a scrofulous taint of body.

For many months preceding her last illness, she had suffered much from anxiety, and various distressing mental emotions.

It was about the beginning of December, 1836, that she began to experience a cough, which she attributed to exposure to cold, caught a few days before. General constitutional disturbance, indicated by anorexia, weakness of the limbs, and a restlessness of the whole system, succeeded. On the following week, it was remarked that the hair fell off in great quantities, and that she was constantly complaining of chills, and of flying transitory pains in different parts of the chest.

During Christmas week, all the unpleasant symptoms were aggravated; she felt so weak that she could not walk about without staggering, and experiencing a sense of painful dizziness; her appetite was completely gone, and she generally rejected by vomiting whatever food she attempted to take; and, lastly, the cough and expectoration—the sputa being still of a yellowish-grey colour, and free from all admixture of blood—had increased. As already mentioned, she took to bed on the last day of the year; and, from this date, the disease ran on in a most rapid course, for between three and four weeks, to its fatal termination.

During the first week of January, the cough was violent and very frequent; the sputa were opaque, dense, and slightly streaked with blood; the breathing was attended with a plaintive moaning sound; the sleep was restless and disturbed with feverish dreams; and she was occasionally slightly delirious, when she first awoke in the morning.

Dr. d'Espine saw the patient for the first time on the 9th. Her breathing was then hurried and distressed; the sound, which attended it, was rather moaning than rough or noisy; the cough was very frequent and troublesome; the sputa were of a thick consistence, yellowish colour, and tinged with dark blood; and the pulse was rapid, but soft and regular. She answered questions with difficulty, complained of humming noises in her ears, and said that it was rather an indescribable uneasiness than any positive pain, that she felt in her chest.

On auscultation, the respiratory murmur was heard over all points of the chest, accompanied at certain places with a vesicular expiration; no blowing nor crepitating bruit was audible; neither was there any bronchophony; but a sonorous and mucous râle was heard occasionally here and there. Although the patient had by this time lost a good deal of flesh, she was far from being much emaciated.

During the next eight days, under a mild antiphlogistic regimen, the cough and expectoration abated in severity and frequency; but some of the other symptoms, such as the inward anxiety about the præcordia and the restless agitated sleep, appeared to be worse than before. She said that she suffered very little pain anywhere; and yet every part of her frame seemed uneasy; the breathing was very frequent, and the pulse retained its former rapidity. Auscultation — which however could never be very minutely performed, in consequence of the restlessness of the patient, and her inability to sit up for any length of time—afforded the same phenomena as on the first examination: the expiration was vesicular at several points, the respiratory murmur "un peu rude," the sonorous râle less strong and distinct, and there was little or no blowing, crepitating, or bronchophonic sound perceptible anywhere.

On the 18th of the month, the patient complained much of tenderness in the left hypochondriac region, and, on examining this part, the spleen was felt to be unusually prominent. In consequence of this symptom, added to certain epigastric phenomena which had recently attracted attention, the idea came across our mind, says M. d'Espine, that the disease of our patient was of a typhoid character; it being difficult to account for the gravity of the symptoms altogether by a simple pulmonary catarrh.

From the 18th to the 25th of the month—the day preceding her death—the chloruret of lime was administered both by the mouth, and also in enemata.

[This practice was no doubt suggested by the idea of the case being typhoid.] —*Rev.*

The cough became less troublesome, and the expectoration less copious; but the breathing was still more hurried and oppressed than before, the pulse was as rapid as ever, and now the lips and angles of the mouth were covered with dark-coloured sordes, and the tongue was dry and parched, as we see it in cases of malignant fever. The thirst was as distressing as ever; the abdomen was tender, especially in the left hypochondriac region; the delirium recurred at uncertain intervals; and the general exhaustion became greater and greater. The pulse rose to 140; the dyspnœa was so great that the poor girl was forced to sit up constantly in bed, in spite of her extreme weakness.

On the morning of the 26th, the distress of breathing was excessive; the face was livid, as we see it in threatened asphyxia; and the general inward suffering was altogether most agonizing to witness. She was happily relieved of all her misery about six o'clock in the evening.

*Dissection.*—The body retained a considerable degree of embonpoint.

Both lungs were found to be adhering, throughout almost their entire extent, to the ribs so firmly that, in removing them from the thorax, a great portion of the pleura costalis was torn off.

The lungs presented an uniform violet-red colour: they did not crepitate when pressed between the fingers, nor did they convey the sensation which hepatised or tuberculated lung does. Upon making a long incision through the entire depth of their substance, the pulmonary tissue exhibited a deep livid hue, mottled with numerous points of tuberculous matter. The tubercles varied in size from that of a grain of rice to about half the size; they were of a yellowish opaline colour, and of so firm a consistence that the scalpel quite grated against them; and they could not be squeezed together between the fingers. The whole substance of the lungs was occupied with them, so that there was scarcely half an inch of extent in any part that was free from them.

They were, however, less numerous, and also of a smaller size and of a less decided colour, as we traced them down from the apex towards the base of the lungs. The interposed cellular substance was every where congested; but this morbid appearance also was not so decidedly marked at the base, as at the superior portions, of both lungs. At no part, however, was the pulmonary substance hepatised; nor did it any where exude on pressure any purulent mucosity.

The two lungs appeared to be, in every respect, similarly affected with the disease.

The other viscera of the body were found to be nearly quite healthy. Even the spleen, which had been suspected during life, was only "assez volumineuse," but not sensibly softened in texture.

*Remarks.*—Judging from the symptoms in the preceding case, a physician could scarcely have been led to have suspected any other disease but, in the early stage, a severe febrile catarrh, threatening to be converted into a pneumonia; and, in the more advanced stage, a typhoid affection associated with bronchitic distress.

Now the post-mortem examination has most clearly disproved the existence of the latter malady—we mean the typhoid affection—as the intestinal mucous glands were found at every part of the gut to be quite sound; and, on the other hand, it has convinced us that all the symptoms were entirely referable to the rapid development of numerous tubercles, throughout almost every part of both lungs. It was, in short, a most genuine case of that form of pulmonary phthisis which has been designated *acute phthisis*, and which is characterised by great constitutional disturbance and a general febrile movement of the whole system, from the time at which the more severe thoracic symptoms set in until the death of the patient.

It is one variety of what has been vulgarly called "galloping consumption." The symptoms never fairly abate, far less cease, for any considerable length of time. The breathing becomes more and more distressed, the pulse is uniformly quickened, the sleep is restless and disturbed with dreams or with delirium, the appetite fails, and every function of the body is more or less deranged. On dissection, the lungs are usually found studded with numerous crude and here and there partially softened tubercles, the intervening interlobular tissue being congested with dark-coloured blood.

The other form of "galloping consumption" is that, wherein a bronchitic attack supervenes on tubercular phthisis, when the tubercles have advanced to softening and suppuration, or perhaps vomicæ have already been formed. The inflammatory action of the mucous lining of the air-passages, coming on in such a diseased state of the lungs, gives rise to a series of symptoms, similar somewhat to that described as characteristic of the first-mentioned form, and in general very speedily proves fatal.

After these preliminary observations, M. d'Espine proceeds to give the following analysis of four of the cases of acute phthisis, detailed in M. Louis' work.

*Duration.*—The difficulty of determining this point satisfactorily, arises from the uncertainty of the precise data, at which the disease may be said to commence. We need scarcely say that the tubercles are already formed before the series of symptoms, which we have described above, commences.

In the preceding case, the period when the patient took to bed (conveniently expressed in the French language by the word *alitement*) was exactly 26 days before the disease terminated fatally. The commencement of the sub-febrile irritation of the system may be dated a fortnight, and that of the cough at least a month, previously. During all this time, however, the patient was able to be going about.

In two of the four cases of acute phthisis reported by M. Louis, the commencement of the severe symptoms was ushered in with a regular febrile attack, and the cough did not supervene till 24 hours in one of the cases, and 10 days in the other, afterwards. In the third case, the commencement was marked by the extreme oppression of the whole system; this was followed by diarrhœa, cough, and pain in the left side; and it was difficult to determine when the regular feverish symptoms might be said to have begun.

In the fourth case, which lasted for 50 days, cough, anorexia, and then pain in the side, ushered in the attack, and the fever was not distinctly appreciable until 11 days subsequently.

If we are to date the commencement of the attack in the case, which we have related above, from the setting-in of the cough, its duration would be 56 days, while in the four cases related by Louis' it was 35, 48, and 50 days. In the remaining case death was induced by an attack of secondary pneumonia, which proved fatal on the 28th day after the beginning of the other symptoms.

*Pathological Anatomy.* — Although the duration of the disease in the preceding case exceeded by a few days that of any of M. Louis' cases, it is remarkable that the lungs were studded only with crude tubercles, and did not exhibit any mass of conglomerated or infiltrated tuberculous matter, nor any trace of softening of their substance, far less of any excavation or vomica.

One only of M. Louis' cases was similar to our's in these respects. It is the fourth one—that which proved fatal on the 28th day, in consequence of the supervention of a pneumonic attack, and the induced hepatisation of a great portion of the pulmonary substance. In this case the lungs were found studded with numerous isolated grey tubercles, which were more numerous, as a matter of course, near the summits than the bases of the lungs.

In the other three cases, portions of the lungs were found infiltrated, to a greater or less extent and degree, with tuberculous matter, softened and partially broken down. In one of these cases, there was a distinct cavern or excavation, similar in every respect to those we see in chronic phthisis, except that it was not lined with false membranes.

It would be an error, however, to conclude from these few cases that the deposition of the tuberculous matter had taken place in the short time, which we have affixed to the duration of the disease. In two or three of M. Louis' cases, there had been a disposition to cough and hurried breathing, although it is expressly stated that the health of the patients had been, previous to the attack, perfectly good.

The most rational inference from all such facts is, that tubercles may be present in the pulmonary tissue for a length of time, and to a great extent, without being necessarily accompanied with very distinct constitutional disturbance.

This circumstance gives rise to many important reflections to the physician. It shews him how cautious he must be in pronouncing an over-confident assurance of no danger in any case, where there is the slightest reason to suspect tubercular disease; although there never has been any well-marked phthisical symptom, such as hæmoptysis, flying pains through the chest, hectic feverishness, or any of the auscultatory signs of the disease present.

We have mentioned, in the account of the dissection in our case, that the pulmonary substance between the various tuberculous deposits was uniformly more or less congested. The same remark applies to M. Louis' cases. The existence of such a state has been very justly considered as a proof of the recent invasion of the disease; as this lesion is seldom present, at least in a very marked degree, in cases of old chronic phthisis.

In three of M. Louis' cases, there were numerous interpleural adhesions.

This excellent pathologist has made a very just remark, in his great work on phthisis, that the thickness and strength of these adhesions are almost always proportional to the gravity of the lesions in the substance of the lungs. Thus, when there are no adhesions, we may be almost certain that we shall not find any excavation within the lungs; when the adhesions are slight, the excavations, if there be any, are almost always small; but, when they are strong and extensive, the excavations are generally numerous, and some of them of considerable dimensions.

The preceding case, however, presents a well-marked exception to the universality of this pathological law, so much insisted on by M. Louis, and which on the whole is, it must be admitted, quite true. It deserves, however, to be remarked, that in our case, the adhesions, although intimate and strong, were not produced by false membranes (il est vrai que l'adherence etait, quoiqu' intime, privée de fausse membrane), but merely by an agglutination of the two opposed serous surfaces.

With respect to the condition of the other viscera in M. Louis' cases of acute phthisis, it is worthy of notice that the intestines were found healthy in all. The spleen, which was "passablement augmentée de volume" in our case, was somewhat enlarged and softened in two of his cases also.

*Symptoms.*—In all M. Louis' cases, the patients suffered from pain at some point of the thorax. Our patient too complained of pain and a sense of tightness from the first day of her "*alitement.*"

Cough was present in all M. Louis' cases; but it differed much in point of intensity and frequency in the different cases. In our patient, the cough, which was most troublesome at about the middle of the disease, diminished so notably at last, that it was very inconsiderable during the eight or ten days preceding death.

The respiration is always very sensibly distressed in genuine acute phthisis. It is usually quickened from the very commencement of the attack, becoming more and more distressed as the disease advances. This is just as we might expect, judging from the post-mortem appearances. The congestion or '*engouement*' of the pulmonary tissue between the tuberculous deposits, must, as a matter of course, offer serious impediment to the free dilatation of the air-vesicles, and must therefore induce the necessity of more frequent efforts to draw in the breath. The frequency of respiration thus increases more and more as the disease advances; and, in its latter stage, it is so painfully urgent, that the poor patient is usually forced to sit up in bed night and day.

The character of the sputa does not afford much assistance in the discrimination of acute phthisis. They are rarely decidedly purulent; but are rather mucous, viscid, opaque, partially streaked with yellow and red lines.

The auscultatory phenomena, too, in this form of phthisis are much less satisfactory than in the more chronic forms of the disease.

In one of M. Louis' cases, all that is said on this subject is merely that, the respiratory murmur was weak in the neighbourhood of one of the clavicles. On dissection, the disease was found not to have advanced further than the stage of granulation-deposit.

In another of the cases, a sonorous râle was heard at first over almost the whole extent of the chest; and latterly a crepitating noise also was perceptible. In the other cases, no mention is made of any distinct sound being audible, save that of an indistinct gurgling at one point, near the upper part of the chest.

M. d'Espine alludes to the peculiarity of the *expiratory bruit*,* as the most characteristic auscultatory phenomenon in his patient, and he expresses his opinion that, had M. Louis been better acquainted with the value of this sign when he reported his cases, he doubtless would have made especial mention of it. It has been only within the last 4 or 5 years that attention has been paid to it. We are mainly indebted for the valuable suggestion of its importance to Dr. Jackson, of Boston.

With respect to the state of the pulse in acute phthisis, the observations of M. Louis quite accord with those we made in our patient's case. It increases in rapidity, as the disease advances towards its final close. In one case it rose to 164; in our patient it did not exceed between 140 and 150.

The profuse perspirations, so invariable a sign of chronic phthisis, were not present in all the cases of the acute form related by M. Louis; neither did they exist, at least to a great extent, in ours. When they do exist, they are generally very limited and partial.

Lastly, with regard to the influence of therapeutic means on the progress of this most fatal malady, *acute phthisis*, we have, alas! only the melancholy part to proclaim the almost total inefficacy of medicine to afford even relief to its most distressing symptoms. There is, perhaps, scarcely any disease which more completely baffles all attempts to arrest or mitigate, far less to subdue, than the acute form of phthisis. The distress of breathing, and the suffering of the whole system, usually increase from worse to worse, in spite of the best directed measures; and the physician has only the melancholy task to watch the gradual aggravation of his patient's sufferings from day to day, without being able to assuage their severity.

In most other acute diseases our art may serve to palliate symptoms, either by depletion, or by derivative means, or lastly by anodynes and soporifics. Not so, in cases of acute phthisis. The inward fire, so to speak, resists all attempts to smother or extinguish it. Certainly none of the remedies, which were employed by M. Chomel in the cases reported by M. Louis, seemed to have any decided effect in affording much relief. Detractions of blood served but to exhaust the vital power, without mitigating the dyspnœa and general distress of the system.

Blisters might relieve the local pain, but they seemed to increase the feverishness, thirst, and irritability.

In M. d'Espine's case the patient seemed to experience partial benefit from a mixture composed of a decoction of polygala senega, syrup of white poppies and tincture of jalap; but, alas! it was

* Hitherto not much regard has been given by auscultators to the *expiratory murmur*, or *bruit*, the attention being altogether directed to that which attends the admission of the air into the chest. The suggestion therefore of M. d'Espine deserves notice; especially in all cases of ambiguity where the usual auscultatory phenomena are indistinct.

deceitful; neither the dyspnœa nor the rapidity of the pulse were at all abated; and even the cerebral symptoms remained as before.

Before concluding our remarks on the history of acute phthisis, let us consider for a few moments its *etiology*.

The age, at which it is usually developed, is between the years of 18 and 22. It has indeed been observed at much later periods of life; but certainly it is much more common about the epoch we have now mentioned, than at any other.

With respect to the sex, as a predisposing cause, we have reason to believe that it is more frequent in young females than males.

What may be considered as rather surprising is, that, in most of the victims of this most destructive form of consumption, the constitution seemed to be, previously to the attack, rather strong and even robust, than very frail and delicate, such as we observe in most other phthisical patients.

It has also been observed that many of the cases of acute phthisis have occurred in subjects, in which the hereditary predisposition to consumption either was inconsiderable, or did not exist at all. M. d'Espine says of his patient, that neither her parents nor any of their other children had shewn any sign of the disease.

M. Louis has remarked in reference to his patients that none of them had suffered from any severe malady beforehand, and that in all of them the health had been generally good. But we should not trust too much to such an observation.

It is very probable that mental disquietude has often a very marked influence, if not on the development, certainly at least on the progress, of acute phthisis.

Disappointment, chagrin, blighted hopes, &c., have often served to awaken the dormant spark, which, when once inflamed, bursts forth with such rapidly destructive power. It is a singular, but well-ascertained fact, that the powers of the mind and, often too, the higher and more amiable feelings of the soul, are very generally developed at an early period of life and with uncommon force and brilliancy in those, who fall the victims of consumption. Any violent excitement, whether it proceeds from joy or grief, disappointment or over-ardent expectation, from intense application or any long-continued effort of the mind, is frequently the more immediate cause of the fatal attack.—*Archives Générales.*

---

### Commentaries on some of the Aphorisms of Hippocrates.

*Section 1st.*

M. Guerbois, one of the surgeons of La Charité Hospital in Paris, and member of the Royal Academy of Medicine, has recently published a French translation of many of the Aphorisms of Hippocrates, appending, to each, commentaries and critical remarks. We shall select a few of the most interesting and instructive.

1. *A very low and scanty diet, continued for a length of time, is always to be avoided in protracted chronic diseases, and even in those that are acute, after the early symptoms are over.*

This Aphorism is quite true; and its truth is duly appreciated by the experienced practitioner. In surgical cases more especially, such as severe accidents, wounds, &c., the low-diet system should not be persevered in beyond the first few days. Nature requires to be supported by mild and nourishing food and sometimes also by cordial stimulants, when she is called upon to repair any serious lesion. Much indeed depends upon the temperament and special constitution of the patient. The truly wise surgeon regulates his practice by a careful study of each case. Of many patients suffering from the same disease, one may be benefited by porter and animal food given daily, another by a lighter diet and wine, a third by the use of quinine or some vegetable aromatic bitter, a fourth by cordial antispasmodics, such as ammonia, valerian, camphor, and opium, &c. As a general remark, aged and very young persons require a more nourishing and cordial regimen, when there is a copious suppuration for a length of time, than those in the more mature periods of life.

Attention to the diet, under such circumstances, is of first-rate importance; and the skilful surgeon will strive to counteract all of the difficulties, which may beset each individual case. M. Guerbois very justly adds; "but every attempt is sure to fail, if he does not make himself master of the imagination of his patient, and exercise a pleasing dominion over his mind." The importance of this conduct is well known to the military surgeon. All wounds are more troublesome of cure in defeated than in victorious troops, in officers than in private soldiers. Gloom, disappointment, and regret, will often cause and keep up a feverish irritability of the system, totally incompatible with the healing process.

3. *The seasons of the year, and the medical constitutions of the atmosphere have a great influence on the prevalence, as well as on the curability, of certain diseases. In judging of the crisis of these diseases, we should attend not only to these circumstances, but also to the condition of the various excretions from the body.*

Those individuals, whose constitutions are naturally weak or have been enfeebled from any cause, physical or mental, are peculiarly susceptible of the influences of the atmosphere and of the seasons.

Each season has, as it were, its special morbific agency. Eruptive fevers are most prevalent in Spring; gastric disorders of all sorts in Summer; agues, &c. in Autumn; catarrhs, pleurisies, &c. in Winter.

Now almost all patients, labouring under surgical complaints, especially such as are attended with profuse suppuration, are more liable to be affected with these prevailing disorders than the sound and healthy are.

The pernicious influence of atmospheric influence on surgical patients is perhaps more distinctly traced in Autumn, than in any other season of the year. In military hospitals this remark is most conspicuously true.

The cause no doubt of this increase is, that the soldiers have probably been exposed during the Spring and Summer months to the hardships and privation of the campaign. Hence dysenteries, typhoid fevers, &c. are usually most frequent and destructive, in camp hospitals, from August to October. The free action of the skin, and the abundant perspiration from it, during the Summer, are probably the causes of the superior health of the soldiers at this time of the year. Doubtless, too, we should take into account the circumstance of the fruits and other vegetable productions not being yet ripe.

4. *Evacuations should not be judged of by their quantity or extent, so much as by the effects which they produce and by the constitution of the patient.*

There is much sound practical wisdom in this aphorism. After severe contusions of the head thorax or abdomen, the very promptest and most active measures are necessary. The great object of the wise surgeon is to prevent the accession of inflammation; and, for this purpose, he employs at once very vigorous and decided measures. Large depletions of blood, and the continued and frequently-repeated use of antifebrile remedies are to be employed immediately.

Here, however, we must enter one *caveat*. When a surgeon is called to a case of severe contusion or other accident, soon after its occurrence, he should be on his guard not to have recourse at once to depleting or depressing means, if the system is still labouring under the shock of the injury. If the pulse be feeble and quick, if the surface is chilly, and the mental powers are bewildered, let him pause for an hour or two, or until Nature has recovered somewhat, and begins to exhibit signs of inordinate re-action. This precaution is especially necessary to be inculcated on the surgeon, in cases of injury of the head. Hitherto, it has been too much the practice with many medical men to whip their lancet out of their case, whenever they are called to a man who has been stunned by a fall or blow. This indiscriminate system of opening a vein is not only very foolish, but often very hurtful. The vital powers may have been "abattus" by the violence; and, before we have recourse to depletion, it is surely but reasonable to allow Nature to recover herself somewhat.

The application of warmth to the feet and stomach, the administration of a spoonful of any light cordial, and the removal of bandages about the limbs and body, may be all that is necessary to be done for some time. True, there is no harm in merely opening a vein, if our object be to ascertain whether the circulation is going on or not; but then this may, in general at least, be discovered by the pulse at the wrist, or at all events by applying the ear over the heart. This simple expedient — the auscultation of the heart's action after all severe accidents—should never be omitted; it will afford us conclusive evidence how the great function of central circulation is going on.

The same remarks hold good in reference to severe contusions, and other injuries, of the abdominal viscera. Every surgeon knows that, in such cases, the liver or spleen is not unfrequently ruptured. Although such an accident is almost inevitably fatal, yet there is no doubt that death will be very much accelerated by bleeding or other depletory measures.

In all accidents occurring in females it is proper to ascertain, at first, the existence or not of pregnancy. In most cases of a severe nature, the fœtus will have been so much injured, that miscarriage is almost inevitable.

5. *Frequent purging is to be avoided in acute diseases, even in their early stages.*

This precept is more applicable to medical than surgical diseases. In reference to the latter, it is quite true.

Not only is the disturbance, caused by frequent evacuations of the bowels, very hurtful after wounds and other injuries, but the weakness also thus produced, and the derivative effects on the bowels, seriously interrupt Nature in her restorative efforts.

We should always remember that, after any severe injury, when there may follow copious and protracted suppuration, or when there may be an extensive destruction of parts, and consequently a large demand on the formative powers of the system, all large drainings of the fluids should be avoided.

English surgeons have erred much more in this particular, than the French.

If the object be to keep down any excess of action in the system, while at the same time the surgeon is anxious to husband, as far as possible, the strength of his patient, perhaps the best plan is to employ the tartar-emetic in small and frequently-repeated doses.

### SECTION II.

6. *In every disease in which the sleep is restless and uneasy, our prognosis must be unfavourable; on the contrary, when the sleep is calm and refreshing, we may augur well of the event.*

This aphorism is applicable especially to wounds and injuries of the head.

In the case of a severe contusion or concussion, if the patient, when he recovers from the stunning effects of the injury (and these may have lasted from a few minutes to several hours or even days), be composed, his intellect clear, and his *physique* calm and temperate, the surgeon will be led to judge favourably of the issue. On the contrary, if the patient, after the cessation of the stupor, remains bewildered and

Hippocrates has very justly remarked, that, when delirium and other such symptoms cease during sleep, we have reason to augur favourably of the case.

7. *General weariness, coming on spontaneously, usually indicates the approach of some malady.*

If a patient, who has sustained a severe wound or other injury, begins, a few days afterwards, to experience great lassitude, pains in all the limbs, tendency to shivering, anorexia, and sleeplessness, let the surgeon's attention be immediately directed to discover what is the cause of these symptoms, and whether any internal viscus is suffering. Even if he fails to detect such, he will do well to act on his guard; to remove all stimulating food, to administer diaphoretics, and perhaps also light laxatives, &c.

The insidious approach of visceral disease, after severe accidents or operations, is well known to every experienced surgeon. Hence the necessity of a most guarded prognosis in all such cases. The surgeon should, for his own sake, act as if he were to be cross-examined afterwards by a troublesome barrister—so unreasonable often is the arguing and expectation of relatives.

8. *If a convalescent does not gain strength in proportion to the quantity of nourishment which he takes, it is a sign that he eats too much.*

When a patient, who for some time has been put upon a full nutritious diet, does not gain either flesh or strength, we have reason to suspect that there is some internal complication, which has hitherto escaped notice. Most frequently perhaps it will be found, that the digestion is imperfectly performed, and that the food therefore is not converted into proper chyle. The alimentary canal may be in a state of atony and weakness.

At other times, there may be a disguised inflammation at some one point or another.

These two states require, as a matter of course, very different modes of treatment. The judicious use of light vegetable tonics, with the addition of small doses of the volatile alkali, will be found of speedy utility in the former case; whereas the latter demands the employment of small topical bleedings, and perhaps also mercurial alteratives, &c.

It is quite impossible to point out all the sources of feverish irritation, which may arise during the progress of a severe injury. The brain, the lungs, the heart, the kidneys, &c.—one or more of them together—may become slightly affected, and ultimately very seriously diseased.

Now, if a full diet and a stimulating regimen be persisted in, while the germs of inward disease are developing themselves, the mischief will inevitably be more and more aggravated; and thus a case, which under judicious management might have been carried to a successful termination, may speedily be rendered desperate. More lives are lost by *over*-feeding than by *under*-feeding patients, who have suffered severe injuries.

We may be quite assured that, if the food, which is taken, does not contribute to the nourishment of the body, it must, as a matter of course, be a source of irritation, by remaining crude and undigested in the bowels. In all cases of disease of the bones, cartilages, and other deep parts, the lightest and least irritating food is the best. How often under such circumstances is there a tendency to diarrhœa and irritation of the bowels!

* We have made one or two passing remarks on sleep, as a means of diagnosis in certain diseases of children, in a preceding article—that on Encephalic irritation.—(*Rev.*)

Such a state must necessarily be aggravated by injudicious food taken into the stomach.

9. *The night, which precedes the crisis of a disease, is usually very restless and uneasy.*

When a wound is very large, as after an amputation of a large member, it has been frequently remarked that the fifth or sixth night following is marked by an extreme agitation, and often too by the accession of violent pyrexial symptoms. The process of suppuration is often ushered in in this matter, and may therefore be regarded in some manner as a *crisis*. Certain it is, that when once it is fairly established, and provided it is, and continues to be, of a healthy laudable nature, the act of reparation and healing goes on quickly and steadily.

The effort, which Nature has been making for several days, is now brought to a *crisis*, and the system seems to be tranquillized in consequence.

10. *Predictions are not absolutely certain in any acute disease, either as favourable or unfavourable.*

In the remarks on Aphorism 8, we have already alluded to the necessity of extreme caution in pronouncing an opinion as to the result of a severe wound or other injury. The simplest accident, at certain times and in certain constitutions, will not unfrequently terminate fatally. Acting on this principle, our forefathers used very justly to say, that "there were no TRIFLING wounds of the head, or of the thorax, or of the abdomen." How often does erysipelas supervene on an inconsiderable wound of the scalp, and cause the most extensive, and not unfrequently fatal, mischief! At other times, a blow on the head—which, severe at the time, may have been thought nothing of—will induce a slow and disguised inflammation of the membranes or of the substance of the brain, thus laying the foundation of that incurable disease, *ramollisement*.

Penetrating wounds of the chest and abdomen are, in a similar manner, often followed by most unexpected accidents. Even in the case of the limbs, an inconsiderable wound may be soon complicated with the most alarming symptoms; such as spasms and tetanus. It is, however, unnecessary to dwell longer on this subject.

The cautious practitioner will never allow himself to talk too lightly of any injury or malady. Independently of the considerations, which we have alleged above, there is another which deserves to be noticed. It was thus aptly put by a young friend, when alluding to a celebrated surgeon, who was supposed to pay little attention to the common run of cases in practice, and devoted himself chiefly to the study of the more serious and complicated diseases. "Mr. —— may think my case a very trifling one, but *I think* it a very serious one." The young practitioner may draw a very useful hint from this passing remark. Nothing so offends a patient as his medical attendant appearing to *think little* of what he himself deems very important.

11. *It is less dangerous when fever succeeds to convulsions, than when convulsions succeed to fever.*

For the first five or six days after the receipt of a severe wound, nervous symptoms, such as startings, and even convulsions, are not unfrequent. These are always to be deemed rather unfavourable signs: often, however, they cease when a febrile paroxysm supervenes, and do not again return; the process of healing now going on regularly and progressively well. In other cases, on the contrary, feverish symptoms set in soon after the injury, and are quickly followed by spasms, delirium, &c. Such cases almost invariably prove fatal. Not more than one case in a hundred of traumatic tetanus recovers. — *La Chirurgie d'Hippocrate, extraite de ses Aphorismes, Paris,* 1837.

[We shall probably continue our notice of this work in our next number. *Rev.*]

